# Localised delivery of interleukin-13 from a PLGA microparticle embedded GelMA hydrogel improves functional and histopathological recovery in a mouse contusion spinal cord injury model

**DOI:** 10.1016/j.bioactmat.2025.07.018

**Published:** 2025-08-08

**Authors:** Ciara M. Walsh, Ruth Colbert, James P. Reynolds, Emily Dunne, Emmanuelle D. Aiyegbusi, Ross O'Carroll, Jacek K. Wychowaniec, Takahiro Masuda, Klaus-Peter Knobeloch, Marco Prinz, Dermot F. Brougham, Dearbhaile Dooley

**Affiliations:** a School of Medicine, Health Sciences Centre, University College Dublin, Belfield, Dublin 4, Ireland; b UCD Conway Institute of Biomolecular & Biomedical Research, University College Dublin, Belfield, Dublin 4, Ireland; c Department of Histopathology, University Hospital Galway, Ireland; d School of Chemistry, University College Dublin, Belfield, Dublin 4, Ireland; e AO Research Institute Davos, Clavadelerstrasse 8, Davos, 7270, Switzerland; f Division of Molecular Neuroimmunology, Medical Institute of Bioregulation, Kyushu University, 3-1-1 Maidashi, Higashi-Ku, Fukuoka, 812-8582, Japan; g Institute of Neuropathology, Faculty of Medicine, University of Freiburg, Freiburg, Germany; h Signalling Research Centres BIOSS and CIBSS, University of Freiburg, Freiburg, Germany

## Abstract

Spinal cord injury (SCI) is a severe neurological condition with limited regenerative capacity and no effective curative treatments. Interleukin-13 (IL-13), an immunomodulatory cytokine, has shown therapeutic potential by promoting alternative immune activation and improving recovery after SCI in mice. However, cell-based IL-13 delivery is hindered by poor graft survival and limited localisation at the injury site. Here, we developed an injectable hydrogel-based delivery system (HGIL13) composed of IL-13-loaded poly(lactic-co-glycolic acid) (PLGA) microparticles embedded in a photocrosslinkable gelatin methacrylate (GelMA) matrix, enabling sustained and localised IL-13 release. HGIL13 achieved IL-13 release for up to six weeks and significantly reduced lipopolysaccharide (LPS)-induced inflammation in BV2 microglia *in vitro*. In a mouse contusion SCI model, HGIL13 enhanced functional recovery, reduced lesion volume, and decreased demyelinated area. Using the Hexb^tdTomato^ mouse we show that HGIL13 modulated the neuroimmune response by decreasing resident microglia density, downregulating CD86 expression, and upregulating Arginase-1 in both microglia and infiltrating monocyte-derived macrophages. RT-qPCR and RNA-seq analyses confirmed sustained immunomodulation over 28 days and indicated early reduction of activated microglia at 7 days post-injury as a key therapeutic mechanism. This study presents a safe, effective, and translatable strategy for localised cytokine delivery, demonstrating strong potential for immunomodulation and improved functional recovery following SCI.

## Highlights

• HGIL13 is an injectable delivery system consisting of IL-13/PLGA microparticles in a GelMA hydrogel.
• HGIL13 releases IL-13 for up to 6 weeks and retains its bioactivity *in vitro*.
• HGIL13 improves functional and histopathological recovery in a mouse contusion SCI model.

## Graphical abstract

**Figure:**
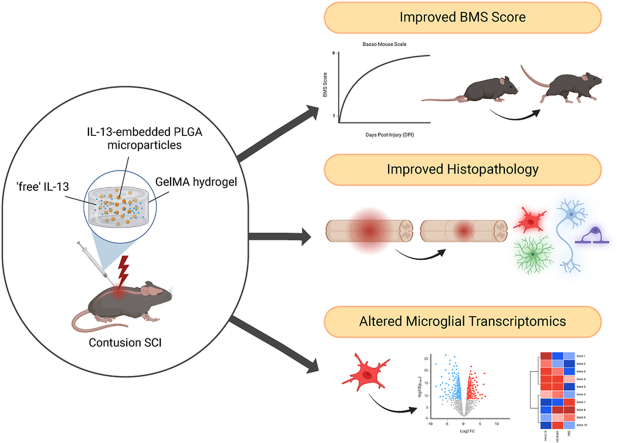

## Abbreviations

Arg-1: Arginase-1
BMS: Basso mouse scale
BSA: Bovine serum albumin
CNS: Central nervous system
CSF1R: Colony stimulating factor 1 receptor
CSPG: chondroitin sulfate proteoglycan
DPI: Days post-injury
DEG: Differentially expressed genes
DMEM: Dulbecco's modified Eagle's medium
DPBS: Dubecco's phosphate buffered saline
ECM: Extracellular matrix
E.E.: Encapsulation efficiency
FBS: Fetal bovine serum
FACS: Fluorescence activated cell sorting
GelMA: Gelatin methacrylate
GFAP: Glial fibrillary acidic protein
GFP: Green fluorescent protein
Iba-1: Ionised calcium binding protein 1
IL-13: Interleukin-13
iNOS: Inducible nitric oxide synthase
LAP: Lithium phenyl (2,4,6-trimethylbenzoyl) phosphinate
LPS: Lipopolysaccharide
LVR: Linear viscoelastic region
MBP: Myelin basic protein
MMP: Matrix metalloproteinase
Padj: Adjusted p-value
PFA: Paraformaldehyde
PLGA: Poly(lactic co-glycolic) acid
P/S: Penicillin/streptomycin
PVA: Polyvinyl alcohol
RFP: Red fluorescent protein
SCI: Spinal cord injury
SEM: Scanning electron microscopy
TGF-β: Transforming growth factor beta
TNF-α: Tumour necrosis factor alpha

## 1 Introduction

Traumatic SCI is a devastating condition that affects hundreds of thousands of individuals worldwide each year [1]. Despite recent progress in pharmacological and surgical interventions aimed at injury management [2,3], there remains no curative treatment for SCI, highlighting the urgent need for novel and effective therapeutic strategies.

SCI pathophysiology is a complex, multiphasic process initiated by the primary mechanical insult to the spinal cord. Upon impact, the primary injury phase lasts for up to 24 h and is characterised by the immediate death of local neural and glial cells. These dying cells release biochemical signals that initiate the secondary injury phase, a prolonged period of cellular and molecular dysfunction that can persist for months post-injury. Secondary injury mechanisms include edema, ischemia, oxidative stress, excitotoxicity, and apoptosis [4,5]. Additionally, the induction of a severe inflammatory response is orchestrated by the infiltration of peripheral immune cells and the activation of local immune cells, termed microglia [6]. Further compounding the injury environment is the formation of a fibrous glial scar around the lesion site by reactive astrocytes, which serves as both a physical and biochemical barrier to axonal regeneration [7,8]. Ultimately the combined events of the secondary injury phase cause damage to propagate away from the primary site of injury and lead to loss of sensorimotor and/or autonomic function in SCI patients.

Due to its extended time course, the secondary inflammatory response is a major therapeutic target in the preclinical SCI landscape [9,10]. Immune cells in the spinal cord can be described as existing on a complex and dynamic spectrum of polarisation ranging from a proinflammatory ‘M1-like’ state to a more neuroprotective ‘M2-like’ state [11]. While this classification is often criticised for its misleading simplicity [[12], [13], [14]], it remains a useful tool when describing immunomodulatory therapeutic approaches whereby immune cells are driven towards an alternatively activated M2-like state to promote regeneration [[15], [16], [17]]. M2-like activation can be further broken down into subtypes ‘M2a’, ‘M2b’ and ‘M2c’, each defined by differential marker expression and different functions in wound healing [18]. M2a cells have increased Arginase-1 (Arg-1) expression and enhanced phagocytic capacity for clearance of apoptotic cell debris, an essential function for creating a permissive microenvironment to allow proliferation of new and existing cells during regeneration [11]. Normally, M2b cells upregulate secretion of various cytokines, including IL-6, IL-10 and tumour necrosis factor alpha (TNF-α), to drive the proliferative phase. IL-10 stimulates M2c polarisation which is defined by increased transforming growth factor beta (TGF-β) secretion to promote angiogenesis and tissue remodelling [18,19]. However, improper activation of M2a cells after SCI leads to chronic inflammation and defective tissue repair. Given the restrictive capacity of M1/M2 terminology for describing complex immune cell polarisation, there is a shift in the field away from this classification towards the use of marker panels and cytokine profiles to better encapsulate the dynamic polarisation state [12]. We heretofore describe the actual microglial/macrophage markers being measured to determine the immune cell state, rather than using the terms ‘M1/M2-like’.

IL-13 is a type-2 cytokine closely related to IL-4, both of which are known inducers of an Arg-1^+^ immune cell response *in vitro* and *in vivo* [[20], [21], [22]]. Endogenous IL-13 expression in the spinal cord is low under normal physiological conditions, and there is evidence to suggest that expression is further decreased following SCI despite the fact that the receptor remains available, rendering IL-13 an interesting therapeutic target [23,24]. Indeed, previous work has shown that IL-13 can drive alternative activation of immune cells in mouse models of SCI, multiple sclerosis and traumatic brain injury [17,[25], [26], [27]]. While recent approaches have shown that cell-based delivery of IL-13 improves recovery after SCI [17,28], the clinical appeal of cell-based drug delivery systems is limited by poor cell graft survival, cellular dedifferentiation and poor localisation at the injury site [[29], [30], [31]].

Biomaterials are emerging as a promising delivery approach due to several advantageous properties, including injectability, biocompatibility and biodegradability [32,33]. These factors facilitate administration directly into the SCI lesion for localised delivery of encapsulated therapeutics that are gradually released over time as the biomaterial degrades, achieving sustained drug delivery. This offers several key advantages over current cell-based and systemic delivery approaches for IL-13 in preclinical studies by achieving sustained and localised cytokine release directly at the lesion site. GelMA hydrogels have been successfully applied for various therapeutic applications in SCI, including cell delivery and tissue engineering [[34], [35], [36]]. We have previously shown that GelMA hydrogels synthesised from type-A gelatin with a translationally relevant photoinitiator can support organotypic spinal cord slice culture *ex vivo* and exhibit low immunogenicity in BV2 microglia and RAW264.7 macrophages [37]. We therefore expect that GelMA hydrogels would provide a suitable platform for the development of a delivery system for immunomodulatory therapeutics in preclinical SCI. One major caveat often observed when using hydrogels for drug delivery is the ‘burst release’ phenomenon whereby encapsulated therapeutics rapidly diffuse out of the hydrogel matrix within 24 h [38]. While this may be useful for acute treatment, the chronic nature of the secondary inflammatory response post-SCI necessitates a sustained delivery approach. PLGA microparticles have been extensively studied for controlled delivery and release of bioactive molecules [[39], [40], [41], [42]]. PLGA microparticles can effectively deliver growth factors and cytokines, protecting them from enzymatic degradation and achieving a sustained therapeutic effect from a single administration [41,42]. We therefore propose a dual-delivery approach for IL-13, whereby IL-13-encapsulated PLGA microparticles are embedded within a GelMA hydrogel matrix with ‘free’ recombinant IL-13 mixed directly into the hydrogel solution prior to crosslinking. We hypothesise that this dual-delivery system, which we have termed ‘HGIL13’, can target both the initial onset of the inflammatory response in the acute injury stage through diffusion-mediated release, with sustained degradation-mediated IL-13 release from the PLGA microparticles to target chronic inflammation.

Differentiating the effect of immunomodulatory therapies on resident microglia versus infiltrating monocyte-derived macrophages has posed a major challenge in the field of neuroinflammation as both cell types are traditionally considered phenotypically indistinguishable [12]. However, recent progress has focused on *Hexb* as a stable microglial-enriched gene that can be used to reliably distinguish between microglia and infiltrating monocytes [43]. Masuda and colleagues developed the Hexb^tdTomato^ reporter line whereby 100 % of microglia throughout the brain and spinal cord are labelled with tdTomato, and tdTomato expression remains stable in models of neurodegeneration and neurotrauma [43]. Therefore, we applied a clinically relevant contusion SCI in the Hexb^tdTomato^ mouse line to fully differentiate and characterise the immunomodulatory effect of HGIL13 on microglia and monocyte-derived macrophages *in vivo*. We show that HGIL13 improved functional and histopathological recovery after SCI by modulating the immune microenvironment at the lesion site. HGIL13 reduced the number of resident microglia, reduced CD86 expression in microglia and increased Arg-1 expression in both microglia and infiltrating monocyte-derived macrophages. Additional transcriptomic analysis of tdTomato^+^ microglia isolated 7 days post-injury (dpi) further highlights the immunomodulatory effect of HGIL13 and points towards an early reduction of the activated microglia population as a potential mechanism of HGIL13-induced repair. Our data provides, to the best of our knowledge, the first description of a translationally relevant biomaterial-based system for IL-13 delivery in SCI. In doing so, we aim to pave the way for innovative and translationally relevant immunomodulatory therapies for preclinical SCI.

## 2 Materials and methods

### 2.1 GelMA synthesis

GelMA was prepared as previously described [37]. Briefly, 10 g gelatin Type A (G2500, Sigma Aldrich) was dissolved in 100 mL DPBS at 60 °C. The temperature was lowered to 50 °C and 5 mL of methacrylic anhydride (276685, Sigma Aldrich) was added at a rate of 0.5 mL/min with gentle mixing. After 1 h, the reaction was quenched by diluting 5-fold in 40 °C Dulbecco's phosphate buffered saline (DPBS). The mixture was then dialysed against 5 L distilled water using 12–14 kDa dialysis tubing (44146, Serva) for 1 week at 40 °C under constant stirring, with a full water change once daily. The solution was lyophilised and stored at 4 °C until further use.

### 2.2 PLGA microparticle synthesis

PLGA microparticles were synthesised using the double emulsion method as previously described [44]. Recombinant murine IL-13 (12340135, Immunotools) was reconstituted according to manufacturer's instructions in 0.1 % bovine serum albumin (BSA). 100 mg PLGA (Resomer® RG 504 H, acid terminated, lactide:glycolide 50:50, M_w_ 38,000–54,000, 719900, Sigma Aldrich) was dissolved in 2 mL dichloromethane (25629.295, VWR) and 20 μg IL-13 in a final volume of 200 μL was added dropwise to this solution in a glass scintillation vial. Blank PLGA microparticles were prepared by substituting the IL-13 solution for an equal volume of 0.1 % BSA. The solution was immediately homogenised at 17,500 rpm for 2 min using the IKA Ultra-Turrax T25 homogeniser to form the primary emulsion. The primary emulsion was then immediately added dropwise to 30 mL 2 % *(w/v)* polyvinyl alcohol solution (PVA, 341584, Sigma Aldrich) in a 50 mL glass beaker, followed by immediate homogenisation at 17,500 rpm for 90 s. The resulting secondary emulsion was stirred overnight at room temperature to allow the dichloromethane to evaporate. Remaining steps were carried out under aseptic conditions. The microparticle solution was transferred to a pre-weighed 50 mL conical tube and centrifuged at 3000×*g* for 6 min at 4°C. Microparticles were washed 3 times by resuspending in 20 mL sterile water followed by centrifugation. After the final wash, particles were resuspended in 1 mL sterile water, frozen at −80°C and lyophilised to obtain a fine dried powder. The tube was reweighed to determine particle yield. Particles were stored at −20°C until use.

### 2.3 IL-13 encapsulation efficiency

Encapsulation efficiency of IL-13 in PLGA microparticles was directly measured by hydrolysing the microparticles in NaOH as previously described [40,45]. Briefly, 5 mg of microparticles were weighed out in duplicate and dissolved in 1 mL 0.1 M NaOH and 5 % SDS overnight on a shaker at 37 °C until a clear solution was obtained. The solution was neutralised by adding 100 μL 1 M HCl and this yielded an aqueous solution that was compatible with ELISA for downstream determination of IL-13 concentration as described in Section 2.7. Encapsulation efficiency was also indirectly determined by measuring the amount of IL-13 remaining in the PVA solution after microparticle synthesis, however the indirect method has been reported to overestimate encapsulation efficiency [46,47] and thus only the direct method is reported here. Encapsulation efficiency (E.E.) was calculated as follows:

$$Direct\;E.E.\;\left(\%\right)=\frac{IL13\;present\;in\;PLGA\;particles}{Total\;IL13\;added}x\;100$$

### 2.4 PLGA degradation *in vitro*

For qualitative assessment of PLGA microparticle degradation *in vitro*, 2 mg microparticles were suspended in 1 mL sterile 1X PBS and incubated at 37 °C on a shaker for 7, 14 or 28 days. At each timepoint, the microparticles were pelleted by centrifugation at 3000×*g* for 5 min, washed once in distilled water, frozen at −80 °C and lyophilised for scanning electron microscopy (SEM) imaging. Particles were mounted on aluminium stubs using carbon-based tape and images were obtained on a Hitachi Regulus 8230 microscope with a voltage of 1000 V for low magnification images and 1400 V for high magnification images.

### 2.5 Hydrogel preparation

Hydrogels were prepared by dissolving GelMA powder in 1X PBS at 3 % *(w/v)* at 37 °C with 0.25 % *(w/v)* photoinitiator lithium phenyl (2,4,6-trimethylbenzoyl) phosphinate (LAP) (900889, Sigma Aldrich). For sterilisation, hydrogel formulations were filtered through a 0.2 μm syringe filter (83.1826.102, Starstedt). PLGA microparticles (blank or IL-13) were mixed with unpolymerised hydrogel solution at 2 % *(w/v)* concentration to obtain HGBLANK or HGIL13 treatments. Resulting hydrogels were photopolymerised using a benchtop visible light source for 2 min (power irradiation was 5.3 W at 1 cm distance from gel).

### 2.6 HGIL13 characterisation

SEM was performed to determine microparticle size and to visualise microparticle distribution within the hydrogel. Briefly, 800 μL HGIL13 was photopolymerised in a 12-well plate as outlined in Section 2.5, lyophilised and SEM imaging was performed as described in Section 2.4 with a voltage of 800 V. Particle diameter and pore size were measured using ImageJ. Hydrogel stiffness with and without microparticles was determined by measuring storage modulus on an Anton Paar MCR301 rheometer fitted with a 25 mm diameter top plate and a 1 mm gap for parallel plate geometry as previously described [88]. An amplitude sweep was initially performed for each polymerised sample to determine the linear viscoelastic region (LVR), at 1 Hz and strain increasing from 0.01 % to 100 % at 37 °C. Then, a frequency sweep was measured from 0.1 to 15 Hz at 0.2 % strain within the LVR of the hydrogels at 37 °C. The storage modulus values shown represent mean ± SEM of 8 samples, taken from frequency sweeps at ω = 6.13 rad s^−1^.

### 2.7 IL-13 release profile

IL-13 release was measured from ‘GelMA’, from ‘PLGA’ microparticles, and from a combination of PLGA microparticles embedded in GelMA, ‘HGIL13’. GelMA samples were prepared by directly incorporating IL-13 at 1000 ng/mL into unpolymerised GelMA, and casting 200 μL of this solution in a 24-well plate for photopolymerisation as described in Section 2.5. PLGA samples were prepared by weighing out 10 mg particles into a 15 mL conical tube. HGIL13 samples were prepared by directly incorporating IL-13 at 1000 ng/mL into unpolymerised GelMA with 2 % *(w/v)* PLGA microparticles. 200 μL of this solution was cast in a 24-well plate and photopolymerised as described in Section 2.5. All samples were prepared in triplicate under aseptic conditions. 2 mL release media (Dulbecco's modified Eagle's medium (DMEM) [LZBE12-604Q, Lonza] supplemented with 0.5 % fetal bovine serum (FBS) [10270106, Gibco], 1 % penicillin/streptomycin (P/S) [15070-063, Gibco] and 20 mM HEPES [H0887, Sigma Aldrich] was gently added to each sample. All samples were incubated at 37 °C with 5 % CO_2_ on a shaker at 88 rpm. At each timepoint (6h, 1d, 3d, 7d, 14d, 21d, 28d, 35d, 42d), release media was collected and replaced, either by gently removing the media from the top of the hydrogel samples or by centrifuging the PLGA samples at 3000×*g* for 5 min to pellet the microparticles. Release samples were stored at −80 °C until use. IL-13 concentration at each timepoint was determined using the Invitrogen Mouse IL-13 Uncoated ELISA kit (88–7137, Thermo Fisher) according to the manufacturer's instructions with the following adaptations. Following plate coating, blocking, and washing, an external standard curve ranging from 31.2 to 1000 pg/mL was prepared by creating a serial dilution of recombinant IL-13 (12340135, Immunotools) in ELISA diluent, and standards and samples were loaded onto the plate and incubated overnight at 4 °C. Following subsequent washing, detection antibody was added and incubated for 2 h at room temperature. Remaining steps were performed as per manufacturer's instructions. Absorbance values were measured on a SpectraMax Plus 384 spectrophotometer at 450 nm with a background wavelength of 590 nm. Results are presented as cumulative IL-13 release per μL of sample.

### 2.8 HGIL13 bioactivity *in vitro*

IL-13 release samples were concentrated using 5 kDa centrifugal filters (NB-57-0001-1, NeoSpin) and the flowthrough was sterilised using 0.22 μm microcentrifuge filters (10310361, Corning). BV2 microglial cells were cultured in DMEM with 10 % FBS, 1 % P/S and 20 mM HEPES. Cultures were maintained in a humidified incubator at 37 °C with 5 % CO_2_. At 70–90 % confluency, cells up to passage 20 were harvested and seeded at 1 × 10^5^ cells/well in a 24-well plate. Cells were starved for 6 h in low-serum media (DMEM, 0.5 % FBS, 1 % P/S and 20 mM HEPES), and then treated for 24 h with either 10 ng/mL LPS (tlrl-b5lps, InvivoGen), 20 ng/mL recombinant (r) IL-13, 10 ng/mL LPS & 20 ng/mL rIL-13, or 10 ng/mL LPS & 20 ng/mL IL-13 released from the HGIL13 system. Treatments were added in low-serum media and after 24 h polarisation markers were measured by qPCR and ELISA as described in Sections 2.9 - 2.10.

### 2.9 RT-qPCR

Total RNA was isolated from BV2 cells using TRIzol reagent (15596018, Thermo Fisher) according to the manufacturer's instructions. RNA was reverse transcribed with SuperScript II reverse transcriptase (18064014, Thermo Fisher). cDNA was amplified on the Applied Biosystems QuantStudio 7 Flex Real-Time PCR System using Power SYBR Green technology (4368706, Applied Biosystems) and the primer pairs outlined in Table 1. Quantification was performed using the ΔCt method with PPIA as a housekeeping gene. Gene expression is displayed as fold change relative to untreated control samples.

**Table 1:** Primer pairs used for RT-qPCR.

Target	Forward primer (5′–3′)	Reverse primer (5′–3′)
Arg-1	GTGAAGAACCCACGGTCTGT	GCCAGAGATGCTTCCAACTG
CD206	GTGGACGCTCTAAGTGCCAT	GAATCTGACACCCAGCGGAA
GFAP	AAGGTCCGCTTCCTGGAA	GGCTCGAAGCTGGTTCAGTT
IL-1β	TGCCACCTTTTGACAGTGATG	ATGTGCTGCTGCGAGATTTG
IL-10	ACAGCCGGGAAGACAATAAC	CAGCTGGTCCTTTGTTTGAAAG
iNOS/Nos2	CAGATCGAGCCCTGGAAGAC	GTGAAGCCATGACCTTTCGC
MBP	GCCTCCGTAGCCAAATCC	GCCTGTCCCTCAGCAGATT
TNF-*α*	AGCCGATGGGTTGTACCTTG	ATAGCAAATCGGCTGACGGT
PPIA	CGTCTCCTTCGAGCTGTTTG	CACCACCCTGGCACATGAAT

### 2.10 ELISA

TNF-α secretion from BV2 cells in Section 2.8 was measured using the DuoSet ELISA system according to the manufacturer's instructions (DY410, R&D Systems). Absorbance values were measured on a SpectraMax Plus 384 spectrophotometer at 450 nm with a background wavelength of 590 nm.

### 2.11 In vivo experiments

All *in vivo* experiments were performed on 8–10 week old wild type C57BL/6J mice or Hexb^tdTomato^ mice derived from a C57BL/6N background [43]. Equal numbers of male and female mice were used. All animals were housed in a specific pathogen-free animal facility at University College Dublin under regular conditions, *i.e.* in a temperature-controlled room (20 ± 3 °C) on a 12-hr day-night light cycle and with food and water *ad libitum*. Procedures involving the use of animals were approved by the Animal Research Ethics Committee at University College Dublin and the Health Products Regulatory Authority of Ireland in accordance with the European Union Directive 2010/63/EU and S.I No. 543 of 2012.

### 2.12 SCI induction and treatment

Animals were randomly divided into 3 groups: ‘PBS’ (PBS injection), ‘HGBLANK’ (3 % (*w/v*) GelMA hydrogel with 2 % (*w/v*) blank PLGA microparticles), or ‘HGIL13’ (3 % (*w/v*) GelMA hydrogel with 1000 ng/mL rIL-13 & 2 % *(w/v)* IL-13/PLGA microparticles). For RNA-sequencing experiments, non-injured ‘Sham’ animals received a laminectomy with no contusion injury. Animals received a perioperative dose of buprenorphine (0.03 mg/kg), and a contusion SCI was performed as follows; animals were anesthetised through inhalation of 5 % isoflurane in an air/oxygen mixture, and anaesthesia was maintained with 1–2 % isoflurane throughout the procedure. Mice were shaved and disinfected with iodine solution, and an incision was made in the skin on the dorsal midline at the thoracic level. A laminectomy was performed at the T8-10 vertebral level, and a contusion injury was induced using the PinPoint™ Precision Cortical Impactor™ (Hatteras, USA) with a flat cylindrical tip diameter 1 mm, velocity 1.5 m/s, depth 1.7 mm, and dwell time 85 ms. Immediately after injury, 1.5 μL of either PBS, HGBLANK or HGIL13 treatment was injected at a depth of 1 mm directly into the lesion, 1 mm rostral to the lesion, and 1 mm caudal to the lesion using a 10 μL gastight syringe (1701 RN, Hamilton) equipped with a 31G bevelled needle. For visualisation of microparticle distribution in the lesion, PLGA microparticles were substituted with FITC-labelled dextran microparticles of an equivalent size (72439, Sigma Aldrich). Injections were performed at a rate of 0.5 μL/min, and the needle was subsequently kept in place for an additional 1 min to allow pressure equilibration and prevent backflow of the injected solution. Following the injections, the muscles and skin were sequentially sutured, animals were injected with glucose (300–500 μL, intraperitoneal) and kept in a heated chamber at 35 °C until recovered from anaesthesia. Buprenorphine (0.03 mg/kg) was administered every 6 h for 72 h as postoperative analgesia. Bladders were manually expressed at least once daily until bladder function returned.

### 2.13 Locomotor function analysis

Starting from 1 dpi, functional recovery was measured using Basso Mouse Scale (BMS) scoring [48]. Two investigators blinded to the experimental groups assessed locomotor function in an open field until the experimental endpoint. Animals were excluded from further analysis if the BMS score was ≥3 at 1 dpi as this was indicative of an insufficiently severe injury. A total of 5 animals were excluded based on this criterion.

### 2.14 Immunofluorescence staining and analysis

At 28 dpi, Hexb^tdTomato^ mice received a lethal dose of sodium pentobarbital and were transcardially perfused with 1X PBS followed by 4 % paraformaldehyde (PFA). Spinal cords were removed and post-fixed overnight in 4 % PFA. Spinal cords were then cryoprotected by sequential overnight incubation in 10 % and 30 % sucrose solution, embedded in optimal cutting temperature matrix (15212776, CellPath) and frozen at −80 °C. Series of 15 μm sections were longitudinally cut on a Leica cryostat and mounted on SuperFrost Plus slides (10149870, VWR). For glial fibrillary acidic protein (GFAP) and myelin basic protein (MBP) staining, slides were blocked in 5 % protein block (ab64226, Abcam) & 0.1 % Triton-X (306324N, BDH Lab Supplies Ltd) in 1X PBS for 30 min. Primary antibodies (Table 2) were incubated overnight in 1 % protein block & 0.05 % Triton-X at 4 °C. For ionised calcium binding protein 1 (Iba-1), CD86 and Arg-1 staining, slides were blocked in 3 % BSA & 0.1 % Triton-X for 30 min, and antibodies were diluted in 1 % BSA. Following 3 washes in 1X PBS, secondary antibodies (Table 2) were incubated for 2 h at room temperature in the appropriate antibody diluent. Slides were washed and counterstained with 300 nM Hoescht 33342 for 10 min at room temperature, followed by 3 washes in 1X PBS and 1 wash in distilled water. Coverslips were mounted with Fluoromount (F4680, Sigma Aldrich).

**Table 2:** Antibodies used for immunofluorescence.

Target	Host	Code	Supplier	Dilution
Arginase-1	Rabbit	ab91279	Abcam	1:300
CD86	Rat	14-0862-82	Thermo Fisher	1:500
Cleaved caspase-3	Rabbit	9661	Cell Signalling Technology	1:200
GFAP	Mouse	G3893	Sigma Aldrich	1:500
Iba-1	Rat	MA5-38266	Thermo Fisher	1:400
Iba-1	Rabbit	MA5-36257	Thermo Fisher	1:400
MBP	Rat	MAB386	Merck	1:250
NeuN	Mouse	94403	Cell Signalling Technology	1:500
Anti-mouse 488	Goat	A32723	Thermo Fisher	1:400
Anti-rabbit 488	Goat	A11008	Thermo Fisher	1:400
Anti-rat 488	Goat	A11006	Thermo Fisher	1:400
Anti-rabbit 647	Goat	A32733	Thermo Fisher	1:400
Anti-rat 647	Goat	A21247	Thermo Fisher	1:400

Slides stained for GFAP or MBP were imaged on an Olympus BX51TF-5 epifluorescence microscope equipped with a Kiralux 5.0 MP Colour CMOS Camera (CS505CU, Thorlabs). To measure lesion size and demyelinated area, 3-7 sections per animal containing the lesion centre were analysed. Using ImageJ FIJI software (NIH), lesion size was defined by manually tracing the lesion border based on GFAP staining while demyelinated area was defined by manually tracing the area devoid of MBP staining as previously described [17,28]. Astrogliosis was assessed using semi-automated ImageJ macros to measure GFAP intensity within 100 μm × 100 μm squares extending 600 μm rostral to 600 μm caudal from the lesion epicentre as previously described [17,28], and within a 100 μm region around the border of the lesion. Microglial infiltration and microgliosis were assessed by measuring tdTomato intensity within 100 μm × 100 μm squares extending 600 μm rostral to 600 μm caudal from the lesion epicentre, and directly in the lesion epicentre using a region that was defined based on corresponding GFAP images. For cleaved caspase-3/NeuN, Iba-1/Arg-1, and Iba-1/CD86 staining, images were acquired on an Olympus FV3000 confocal microscope. To quantify neuronal and microglial apoptosis, 3-7 sections per animal were analysed by counting cleaved caspase-3^+^NeuN^+^ cells and cleaved caspase-3^+^tdTomato^+^ cells in two 10X images covering the entire lesion site using CellProfiler software [49]. To quantify immune cell polarisation, 3-7 sections per animal were stained for either Iba-1 & CD86, or Iba-1 & Arg-1, respectively. Monocyte-derived macrophages were defined as Iba-1^+^tdTomato^−^ and microglia were defined as Iba-1^+^tdTomato^+^. The overall number and morphology of microglia and monocyte-derived macrophages, as well as the number and intensity of CD86^+^ and Arg-1^+^ microglia and monocyte-derived macrophages were quantified from four 20X images covering the lesion epicentre and the immediate rostral and caudal perilesional area using CellProfiler software. Data from all regions of interest within a section were summed and then averaged across all sections to determine the final value per animal.

### 2.15 RNA and protein extraction from bulk tissue

At predefined timepoints (6h, 1d, 3d, 7d, 14d, 28d), wild-type C57BL/6J mice received a lethal dose of sodium pentobarbital and were subsequently transcardially perfused with ice-cold 1X PBS. Spinal cords were rapidly isolated, and 1 cm length of cord centred around the lesion was snap frozen in liquid nitrogen. Tissue was homogenised using a Qiagen TissueLyzer and processed for RNA using TRIzol reagent or for protein using RIPA buffer (89900, Thermo Fisher) and HALT protease inhibitor (10320015, Thermo Fisher) according to the manufacturer's instructions. IL-13 quantification was performed by ELISA as described in Section 2.7. cDNA synthesis and RT-qPCR were performed as described in Section 2.9.

### 2.16 FACS isolation of microglia from Hexb^tdTomato^ mice

At 7 dpi, Hexb^tdTomato^ mice received a lethal dose of sodium pentobarbital and were transcardially perfused with ice-cold DPBS (14287-072, Thermo Fisher). Spinal cords were rapidly isolated, and 1 cm length of cord centred around the lesion was immediately processed for fluorescence activated cell sorting (FACS) of microglia using the adult brain dissociation kit (130-107-677, Miltenyi Biotec). Briefly, tissue was roughly mechanical dissociated with a small scissors and transferred to 1950 μL Enzyme Mix 1 containing added transcription and translation inhibitors, namely actinomycin D (5 μg/mL, A1410, Millipore Sigma), triptolide (10 μM, T3652, Millipore Sigma) and anisomycin (10 μg/mL, A9789, Millipore Sigma). 30 μL Enzyme Mix 2 was added and samples were dissociated on a shaker at 37 °C for 1 h at 240 rpm. Digested tissue was passed through a pre-wetted 70 μm MACS SmartStrainer (130-098-462, Miltenyi Biotec) and pelleted at 300×*g* for 10 min at 4 °C. Supernatant was aspirated and debris was removed using the adult brain dissociation kit Debris Removal solution according to the manufacturer's instructions. The final cell pellet was resuspended in 1 mL 0.5 % BSA in DPBS, and tdTomato^+^ microglia were sorted directly into 350 μL lysis buffer (buffer RLT Plus from RNeasy Plus Micro kit [74034, Qiagen]) using the CytoFLEX SRT cell sorter. Sorted cells were vortexed thoroughly and immediately stored at −80 °C until RNA isolation using the RNeasy Plus Micro kit according to the manufacturer's instructions.

### 2.17 RNA-sequencing of microglia from Hexb^tdTomato^ mice

cDNA library construction, Illumina sequencing and gene expression quantification were performed by Novogene. mRNA was purified from total RNA using poly-T oligo-attached magnetic beads and amplified by SMARTer technology prior to library prep. The cDNA library was prepared and sequenced on the Illumina NovaSeq X Plus Series (PE150). Raw data were processed through fastp software and mapped to the GRCm39 mus musculus reference genome using Hisat2 software (v2.0.5). The number of reads mapped to each gene was counted using featureCounts (v1.5.0-p3) and gene expression level was calculated as FPKM, expected number of Fragments Per Kilobase of transcript sequence per Millions base pairs sequenced [50].

### 2.18 RNA-sequencing analysis

Differential expression analysis was performed using DESeq2 (v1.42.1) in RStudio (v4.3.2) with Benjamini-Hochberg correction for multiple testing [51]. Differentially expressed genes (DEGs) were defined using the criteria |log2FoldChange| ≥ 1 and adjusted p-value (padj) ≤ 0.05. Gene ontology enrichment analysis of DEGs was performed using the clusterProfiler R package (v4.10.1), with padj ≤0.05. Volcano plots, Venn diagrams and heatmaps of DEGs were generated in R using ggplot (v3.5.1), VennDiagram (v1.7.3) and pheatmap (v1.0.12).

### 2.19 Statistical analysis

All statistical analyses were performed using GraphPad Prism 8.0 software unless otherwise stated. Data were tested for normality using the Shapiro-Wilk test. For analysis of cell morphology and intensity described in Section 2.14, outliers were identified and removed using the robust regression and outlier removal method in Prism with a Q coefficient value of 1 % [52]. Remaining data were averaged per animal. Details of statistical tests and animal numbers used for each experiment are given in the figure legends. Differences were considered statistically significant when p ≤ 0.05. Data shown represent mean values per experimental group ± SEM unless otherwise stated.

## 3 Results

### 3.1 HGIL13 characterisation

**Fig. 1:**
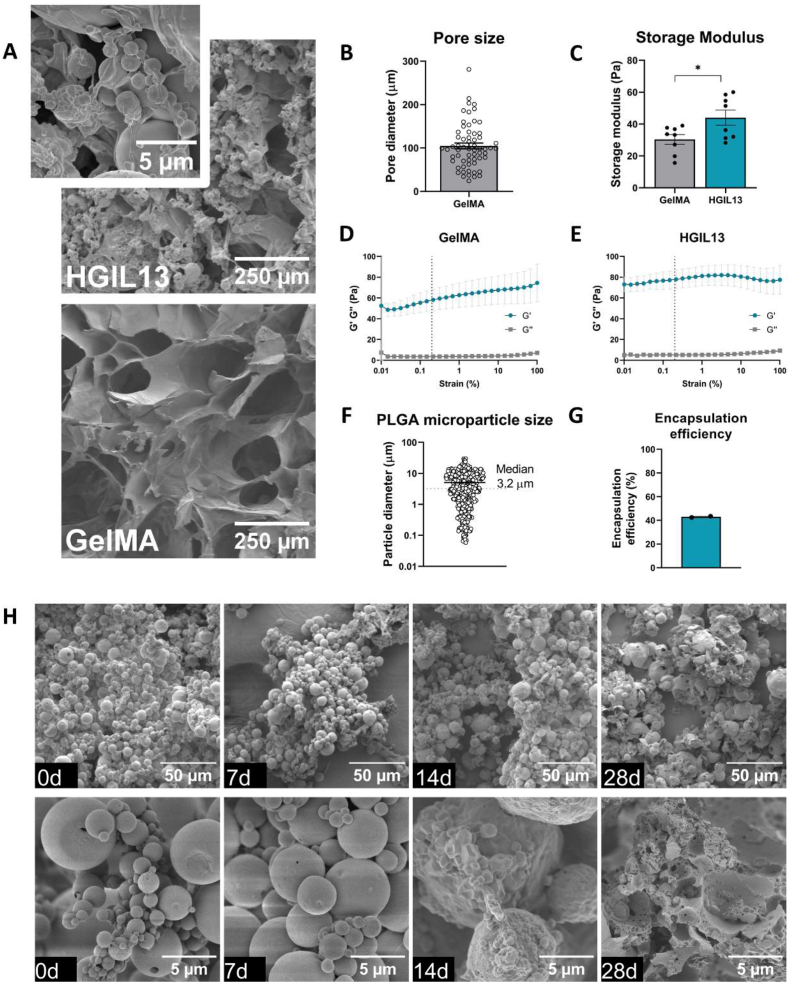
HGIL13 characterisation. (A) SEM imaging of HGIL13 and GelMA hydrogel. (B) Pore size diameter of GelMA hydrogel, n = 62 pores. (C) Storage modulus of GelMA and HGIL13, n = 8 samples/group. Amplitude sweeps of (D) GelMA and (E) HGIL13 with 0.2 % strain within the LVR. (F) PLGA microparticle size distribution, n = 539 microparticles. (G) Encapsulation efficiency of IL-13 in PLGA microparticles. (H) Corresponding low and high magnification SEM images of PLGA microparticles after 0, 7, 14 or 28 days incubation showing physical degradation *in vitro*. Data are presented as mean ± SEM. Analysis in (C) by unpaired Student's t-test, ∗p < 0.05.

SEM imaging was performed to visualise the microarchitecture of the GelMA hydrogel and the surface morphology of PLGA microparticles (Fig. 1A). GelMA had a porous structure with an average pore size of 105 ± 5 μm (Fig. 1A and B). An amplitude sweep was performed to determine the LVR of the hydrogel and hydrogel stiffness was then measured within this region (Fig. 1D and E). The addition of PLGA microparticles to GelMA increased the storage modulus from 30 ± 3 Pa to 44 ± 5 Pa (Fig. 1C). PLGA microparticles displayed a smooth spherical surface with an average particle diameter of 3.2 ± 0.2 μm (Fig. 1F). The average microparticle yield after filtration was 29.4 ± 6.6 %. The encapsulation efficiency of IL-13 in PLGA microparticles was measured by directly quantifying IL-13 that was released during hydrolysis of a known quantity of microparticles and was determined to be 43.0 ± 0.5 % (Fig. 1G). Given that 100 % encapsulation efficiency would yield 200 ng IL-13/mg microparticles (Section 2.2), an encapsulation efficiency of 43 % indicates a total IL-13 content of 86 ng IL-13/mg microparticles. Considering the concentration of ‘free’ IL-13 added to the hydrogel (1000 ng/mL), PLGA microparticles at 2 % (*w/v*) with a concentration of 86 ng IL-13/mg, and an injection volume of 4.5 μL *in vivo* (Section 2.12), this equates to a total dose of 12.2 ng IL-13 per animal. PLGA microparticle degradation was studied by incubating microparticles at 37 °C for various durations and subsequently using SEM imaging to observe changes in morphology. Initially, there were limited signs of degradation after 7 days. However, by 14 days, the characteristic smooth particle morphology began to dimple as the surface was compromised (Fig. 1H). By 28 days, SEM images reveal significant degradation, highlighting the capacity of PLGA microparticles for long-term release of therapeutic cargo during this timeframe.

### 3.2 HGIL13 releases IL-13 for up to 6 weeks and can counteract LPS-induced inflammatory responses in BV2 microglia *in vitro*

**Fig. 2:**
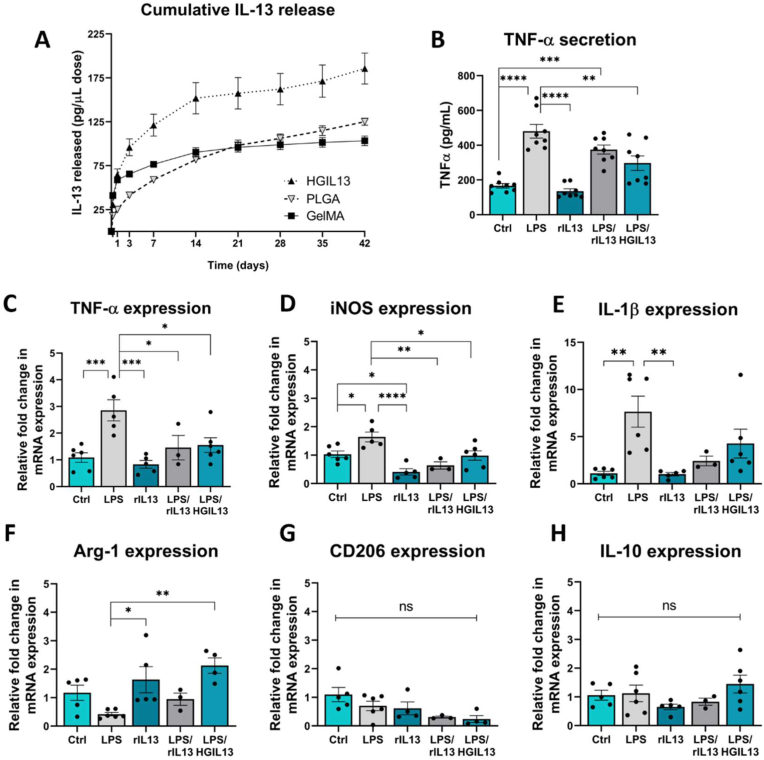
HGIL13 retains bioactivity *in vitro*. (A) Cumulative IL-13 release from HGIL13 (GelMA hydrogel with 2 % (w/v) IL13/PLGA microparticles & 1000 ng/mL IL-13), PLGA (IL-13/PLGA microparticles only, no hydrogel) or GelMA (1000 ng/mL IL-13 in GelMA hydrogel) over 6 weeks *in vitro*, n = 3 samples/group. Released IL-13 was collected and the following culture conditions were established in BV2 microglia to test its bioactivity: (i) Ctrl (no stimulation), (ii) LPS (10 ng/mL LPS), (iii) rIL13 (20 ng/mL recombinant (r) IL-13), (iv) LPS/rIL13 (10 ng/mL LPS & 20 ng/mL rIL-13), and (v) LPS/HGIL13 (10 ng/mL LPS & 20 ng/mL IL-13 released from the HGIL13 system). After 24 h, the expression of (B, C) TNF-α, (D) iNOS, (E) IL-1β, (F) Arg-1, (G) CD206 and (H) IL-10 were measured. Gene expression is represented as fold change compared to control using PPIA as a housekeeping gene. Data represent mean ± SEM of 3 independent experiments. Analysis by one-way ANOVA with Tukey's multiple comparisons test, ∗p < 0.05, ∗∗p < 0.01, ∗∗∗p < 0.001, ∗∗∗∗p < 0.0001, ns p > 0.05.

IL-13 release was measured from HGIL13, from PLGA microparticles and from GelMA hydrogel over 6 weeks *in vitro*. To normalise the release data from these 3 systems, and given the ultimate *in vivo* application of this work, the results presented in Fig. 2A represent the amount of IL-13 released per μL of treatment. Comparable burst release of IL-13 was seen from both HGIL13 and GelMA over 24 h as the IL-13 rapidly diffused out of the hydrogel network. This was followed by limited IL-13 release from GelMA, as demonstrated by the flattened release curve corresponding to depletion of mobile drug (Fig. 2A). In contrast, a sustained release of IL-13 was observed from both HGIL13 and PLGA for up to 6 weeks. The final cumulative amount of IL-13 released from HGIL13 was 186 ± 18 pg IL-13/μL, which did not significantly differ from the overall expected release of IL-13 based on the summed release data from GelMA and PLGA (Fig. S1). The cumulative totals from GelMA and PLGA were 103 ± 6 pg/μL and 125 ± 4 pg/μL, respectively.

IL-13 from each timepoint was collected and added to BV2 microglia in the presence of 10 ng/mL LPS to assess if released IL-13 could counteract an inflammatory response to the same degree as an equal concentration of pure recombinant (r)IL-13, therefore assessing if the bioactivity of IL-13 persisted after release from the HGIL13 system. As expected, the addition of LPS to BV2 microglia caused a significant increase in proinflammatory markers TNF-α, inducible nitric oxide synthase (iNOS) and IL-1β (Fig. 2B–E). Cotreatment with rIL-13 or an equal concentration of IL-13 collected from HGIL13 reduced expression of TNF-α and iNOS back to control levels, indicating that released IL-13 from HGIL13 could counteract the inflammatory effects of LPS to the same degree as rIL13 (Fig. 2B–D). Additionally, cotreatment with LPS and released IL13 from HGIL13 increased Arg-1 expression compared to LPS treatment alone, demonstrating that HGIL13 can reverse LPS-induced responses *in vitro* (Fig. 2F). There was no change observed in IL-10 or CD206 expression in any of the treatment groups (Fig. 2G and H).

### 3.3 HGIL13 improves functional recovery and reduces lesion size and demyelinated area in a mouse contusion SCI model

**Fig. 3:**
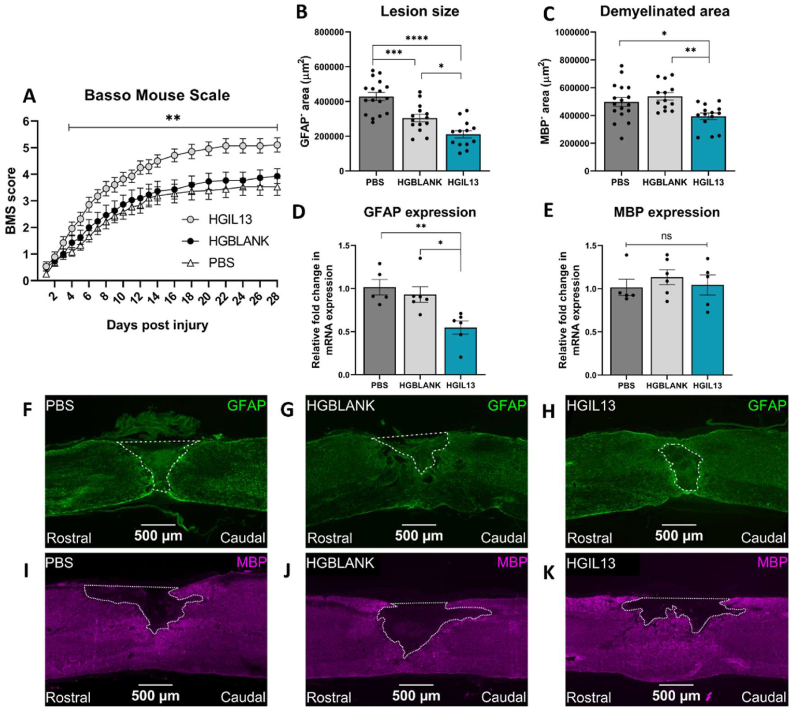
HGIL13 improves functional recovery and reduces lesion size and demyelinated area. (A) BMS scores of Hexb^tdTomato^ mice receiving HGIL13 treatment were increased following SCI compared to controls. Data represent mean ± SEM of n = 14–18 mice/group. Analysis by repeated measures two-way ANOVA with Tukey's multiple comparisons test, ∗p < 0.05 or ∗∗p < 0.01 compared to HGBLANK and PBS groups, respectively. Image analysis revealed a reduction in (B) lesion size and (C) demyelinated area, with corresponding mRNA expression levels of (D) GFAP and (E) MBP at 28 dpi. Gene expression is represented as fold change compared to PBS with PPIA as a housekeeping gene. Representative photomicrographs of Hexb^tdTomato^ spinal cord sections including the lesion epicentre of (F, I) PBS, (G, J) HGBLANK, and (H, K) HGIL13-treated mice. Sections were stained for (F–H) GFAP (green) and (I–K) MBP (magenta) to determine lesion size and demyelinated area as depicted by the dotted white line. Scale bars represent 500 μm. Data represent mean ± SEM of n = 13–17 mice/group. Analysis by one-way ANOVA with Tukey's multiple comparisons test, ∗p < 0.05, ∗∗p < 0.01, ∗∗∗p < 0.001, ∗∗∗∗p < 0.0001.

To investigate the therapeutic efficacy of HGIL13 *in vivo*, Hexb^tdTomato^ mice were treated with either PBS (control group), GelMA hydrogel with empty PLGA microparticles (HGBLANK), or GelMA hydrogel with IL-13 PLGA microparticles and 1000 ng/mL rIL-13 (HGIL13) immediately after contusion SCI. IL-13 was detected in the lesion site of HGIL13-treated mice 6 h post-injury, while visualisation of fluorescently labelled microparticles in the spinal cord at 28 dpi demonstrated the successful localisation of the hydrogel/microparticle mixture within and throughout the lesion site (Fig. S2). Functional recovery was measured up to 28 dpi using BMS scoring, at which point tissue was harvested and immunostained for GFAP and MBP to determine lesion size and demyelinated area, respectively. HGIL13 significantly improved functional recovery from 4 dpi onwards compared to PBS and HGBLANK-treated animals (Fig. 3A). Histological evaluation showed that lesion size and demyelinated area were reduced following HGIL13 treatment (Fig. 3B and C). Gene expression analysis of GFAP and MBP by RT-qPCR showed that GFAP expression was reduced in HGIL13-treated animals at 28 dpi, however no change was observed in MBP expression (Fig. 3D and E). A significant reduction in lesion size was also observed following HGBLANK treatment (Fig. 3B), however there was no effect observed on functional recovery, demyelinated area, or gene expression in HGBLANK-treated animals (Fig. 3C–E). Taken together, these results demonstrate that HGIL13 significantly improves recovery on both a functional and histopathological level after contusion SCI.

### 3.4 HGIL13 reduces astrogliosis and decreases microglial infiltration at the lesion site in Hexb^tdTomato^ mice

**Fig. 4:**
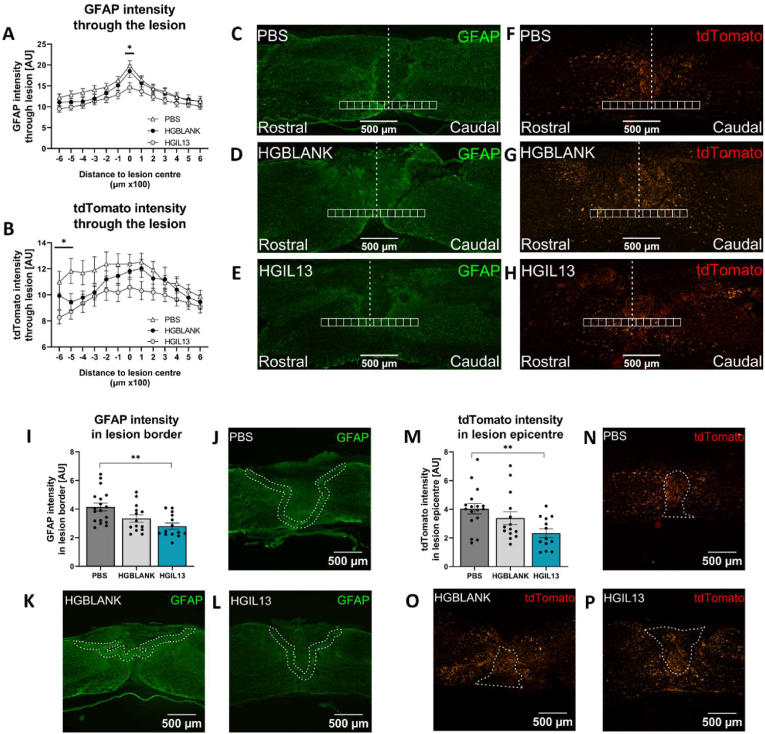
HGIL13 reduces astrogliosis and microglial abundance at the lesion. (A) GFAP and (B) tdTomato intensity analysis in the injured spinal cord of Hexb^tdTomato^ mice at 28 dpi. Representative photomicrographs in (C–H) depicting GFAP (green) or tdTomato (red) and the 100 × 100 μm square regions of interest extending 600 μm rostral to 600 μm caudal from the lesion epicenter. (I) GFAP intensity within a 100 μm perilesional region is significantly reduced following HGIL13 treatment compared to PBS-treated controls. This border region is depicted by dotted white lines in representative photomicrographs (J–L). (M) tdTomato intensity within the lesion is significantly reduced following HGIL13 treatment compared to PBS-treated controls. The lesion was defined based on corresponding GFAP staining and is depicted by dotted white lines in representative photomicrographs (N–P). Scale bars represent 500 μm. Data represent mean ± SEM of n = 13–17 mice/group. Analysis by one-way ANOVA, or repeated measures two-way ANOVA for (A, B), with Tukey's multiple comparisons test, ∗p < 0.05, ∗∗p < 0.01.

Astrocyte reactivity, or astrogliosis, occurs after SCI and is characterised by increased GFAP expression as astrocytes surround the lesion and deposit inhibitory chondroitin sulfate proteoglycans (CSPGs) to form the glial scar [7,8]. Microglia are recruited to the lesion site where they proliferate and contribute to the secondary inflammatory cascade [6]. To investigate the effect of HGIL13 treatment on this pathological glial response after SCI, we analysed GFAP (Fig. 4C–E) and tdTomato (Fig. 4F–H) intensity through the lesion in square regions extending 600 μm caudal and 600 μm rostral from the lesion site. There was a significant reduction in GFAP intensity at the lesion epicentre following HGIL13 treatment compared to PBS-treated controls (Fig. 4A). There was no change in tdTomato intensity at the lesion epicentre, however a reduction was observed 500–600 μm rostral to the lesion site (Fig. 4B). To further investigate astrocyte reactivity in the perilesional area corresponding to the glial scar, we analysed GFAP intensity in a 100 μm region bordering the lesion site (Fig. 4I–L). GFAP intensity was reduced in this region following HGIL13 treatment compared to PBS-treated controls (Fig. 4I). Additionally, tdTomato intensity within the entire lesion was measured as a proxy of microglial abundance in the lesion (Fig. 4M–P). This region was defined based on corresponding GFAP staining. Using this method, we found that tdTomato intensity was reduced within the lesion in HGIL13-treated animals compared to PBS-treated controls (Fig. 4M). Overall, these data indicate that HGIL13 significantly reduces astrogliosis and decreases microglial abundance at the lesion site after SCI.

### 3.5 HGIL13 reduces the number of apoptotic neurons at the lesion site

**Fig. 5:**
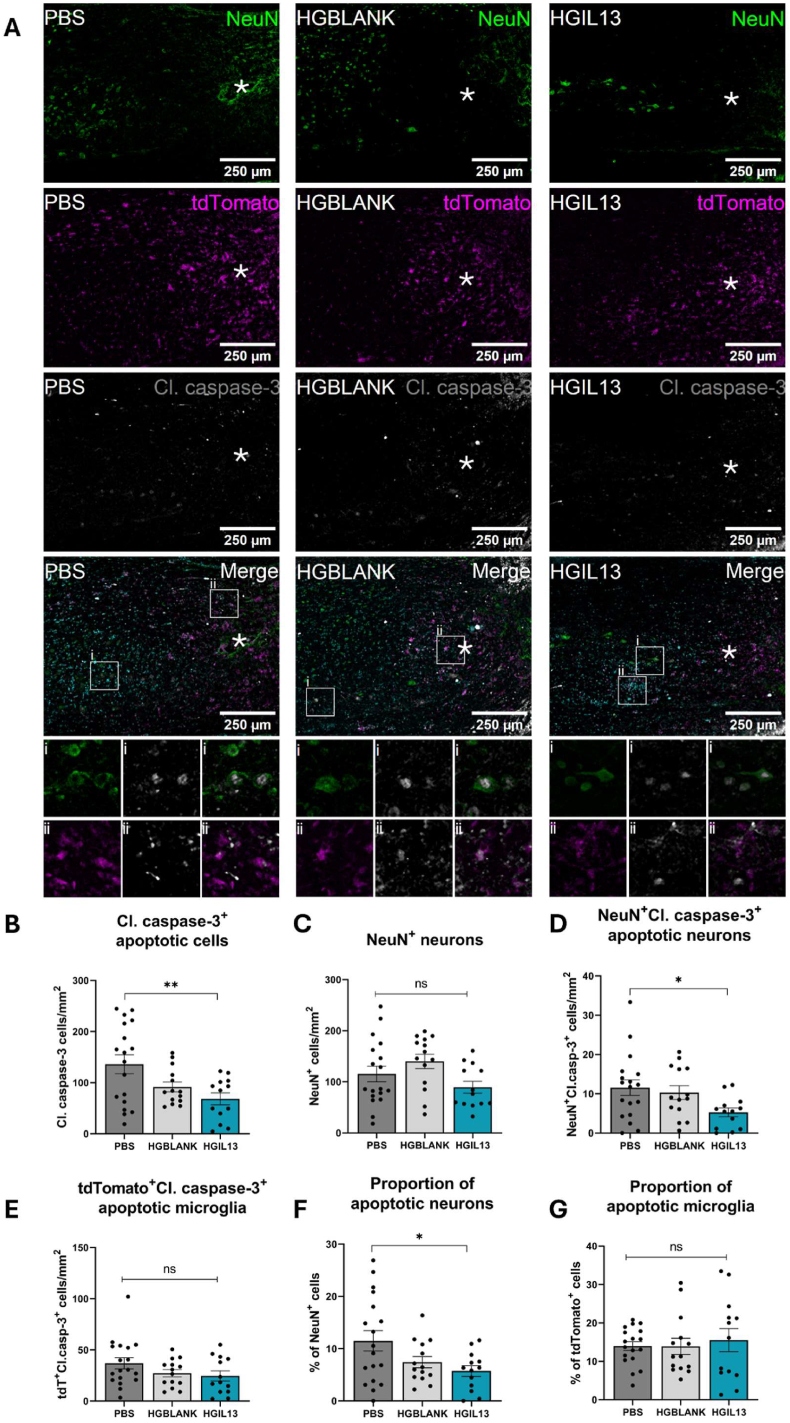
HGIL13 reduces neuronal apoptosis and has no effect on microglial apoptosis. (A) Representative photomicrographs of longitudinal spinal cord sections from Hexb^tdTomato^ mice at 28 dpi depicting neurons (green, NeuN), microglia (magenta, tdTomato) and apoptotic cells (grey, cleaved caspase-3), with details of selected apoptotic neurons and apoptotic microglia outlined in white boxes and shown below. The lesion epicentre is shown by a white asterisk. The number of (B) cleaved caspase-3^+^ apoptotic cells, (C) NeuN^+^ neurons, (D) cleaved caspase-3^+^NeuN^+^ apoptotic neurons and (F) cleaved caspase-3^+^tdTomato^+^ apoptotic microglia were analysed, as well as (F) the proportion of apoptotic neurons or (G) microglia relative to the overall number of each cell type. Scale bars represent 250 μm. Data represent mean ± SEM of n = 13–18 mice/group. Analysis by one-way ANOVA with Tukey's multiple comparisons test, ∗p < 0.05, ∗∗p < 0.01, ns p > 0.05.

Given the improved functional and histopathological outcome following HGIL13 treatment, we then explored the neuroprotective effect of HGIL13 by assessing neuronal apoptosis at 28 dpi using cleaved caspase-3 as an apoptotic marker and NeuN as a pan-neuronal marker (Fig. 5). Our results revealed a significant reduction in the total number of cleaved caspase-3^+^ cells at the lesion site following HGIL13 treatment (Fig. 5B). Although there was no difference in the overall number of NeuN^+^ cells, there was a significant reduction in the number and proportion of cleaved caspase-3^+^NeuN^+^ neurons following HGIL13 treatment (Fig. 5C,D,F). These findings indicate that while HGIL13 treatment effectively reduced neuronal death after SCI, it did not promote neuronal regeneration. Previous studies have suggested that IL-13 exerts neuroprotective effects by inducing apoptosis in microglia, thereby mitigating the harmful impact of reactive microglia on regenerating neurons [[53], [54], [55]]. To investigate whether this mechanism applied in our model, we analysed microglial apoptosis at 28 dpi using cleaved caspase-3 and tdTomato markers. HGIL13 treatment did not affect the number or relative proportion of cleaved caspase-3^+^ microglia at this timepoint (Fig. 5E–G). In summary, our findings demonstrate that HGIL13 possesses neuroprotective properties through its ability to reduce apoptosis in neurons.

### 3.6 HGIL13 reduces the number of microglia at the lesion site

**Fig. 6:**
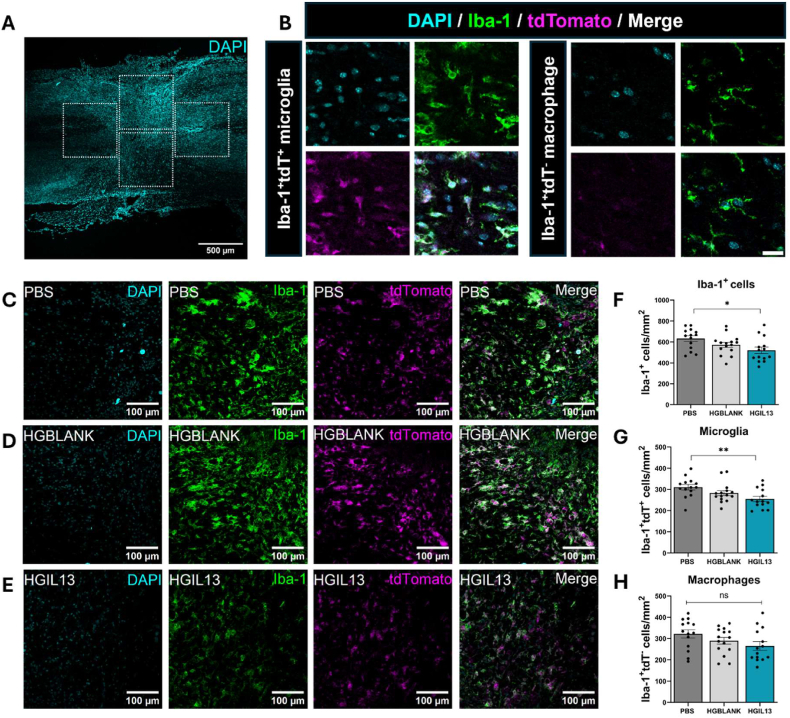
HGIL13 reduces the number of microglia at the lesion site. (A) Overview of the regions used for analysis of microglia and monocyte-derived macrophages in Hexb^tdTomato^ mice, scale bar represents 500 μm. (B) Hexb^tdTomato^ mice have the tdTomato fluorophore (magenta) stably and specifically expressed in microglia. When used in conjunction with a pan-macrophage marker, e.g. Iba-1 (green), this allows for reliable differentiation between Iba-1^+^tdT^+^ microglia and Iba-1^+^tdT^−^ monocyte-derived macrophages. Scale bar represents 20 μm. (C–E) Representative photomicrographs depicting Iba-1 and tdTomato, scale bars represent 100 μm. Quantification of (F) Iba-1^+^ immune cells, (G) Iba-1^+^tdT^+^ microglia and (H) Iba-1^+^tdT^−^ monocyte-derived macrophages shows a significant reduction in the number of microglia following HGIL13 treatment at 28 dpi. Data represent mean ± SEM of n = 14–15 mice/group. Analysis by one-way ANOVA with Tukey's multiple comparisons test. ∗p < 0.05, ∗∗p < 0.01, ns p > 0.05.

Differentiating between resident microglia and infiltrating monocyte-derived macrophages has posed a challenge in the field of neuroinflammation, as both cell types express similar markers and become phenotypically indistinguishable once infiltrating macrophages enter the lesion microenvironment [56]. To investigate the immunomodulatory effect of HGIL13 after SCI, we took advantage of the Hexb^tdTomato^ mouse line and used Iba-1 as a pan-macrophage marker such that double-labelled microglia (Iba-1^+^tdTomato^+^) could be differentiated from single-labelled monocyte-derived macrophages (Iba-1^+^tdTomato^−^) across four regions of interest covering the lesion site (Fig. 6A–B). HGIL13 significantly reduced the overall number of Iba-1^+^ immune cells compared to PBS-treated controls, and this reduction was due to a significant decrease in the number of microglia rather than in the number of infiltrating monocytes (Fig. 6F–H).

We then analysed the morphology of the microglial and macrophage populations within the lesion epicentre, using cell area, circularity, perimeter, and diameter as readouts to determine if the cells displayed an amoeboid morphology or a ramified morphology, which may be indicative of an activated versus a resting surveillant state, respectively (Fig. S3). Typically, an amoeboid cell has a smaller cell area, perimeter, and diameter than a ramified counterpart, leading to higher circularity on a scale of 0–1, i.e. a perfect circle [[57], [58], [59]]. Although an increase in microglial area was observed following HGIL13 treatment, there was no further evidence to suggest that HGIL13 altered the morphology of microglia or monocyte-derived macrophages (Fig. S3D–K).

### 3.7 HGIL13 reduces CD86 expression in microglia and increases Arg-1 expression in both microglia and infiltrating monocyte-derived macrophages

**Fig. 7:**
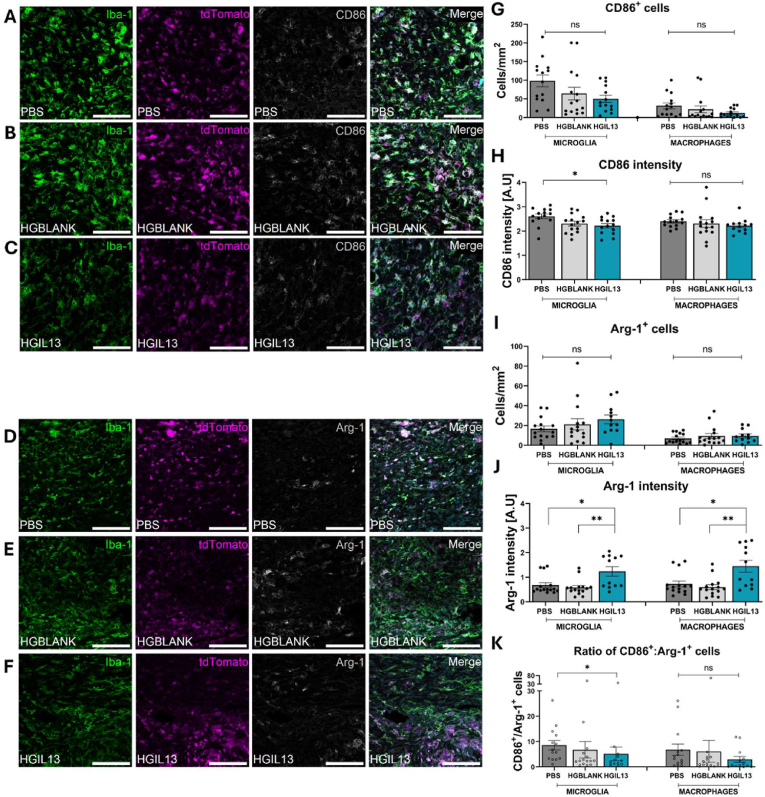
HGIL13 reduces CD86 expression in microglia and increases Arg-1 expression in microglia and monocyte-derived macrophages after SCI. *(A-F) Representative photomicrographs depicting Iba-1 (green), tdTomato (magenta), and (A-C) CD86 or (D-F) Arg-1 (grey) for quantification of immune polarisation in Iba-1*^*+*^*tdT* ^*+*^ *microglia and Iba-1*^*+*^*tdT*^*-*^*monocyte-derived macrophages at 28 dpi in Hexb*^*tdTomato*^*mice. (G) CD86*^*+*^*cell counts, (H) CD86 intensity, (I) Arg-1*^*+*^*cell counts and (J) Arg-1 intensity were quantified using an automated pipeline in CellProfiler as described in* Section 2.14*. (K) The ratio of CD86*^*+*^*to Arg-1*^*+*^*cells was determined by dividing the number of CD86*^*+*^*cells by the number of Arg-1*^*+*^*cells. Scale bars represent* 100 μm*. Data represent mean ± SEM of n = 12-18 mice/group. (G-I) Analysis by one-way ANOVA with Tukey's multiple comparisons test. (J, K) Analysis by Kruskal-Wallis with Dunn's multiple comparisons test. ∗p < 0.05, ∗∗p < 0.01, ns p > 0.05.*

We then applied CD86 or Arg-1 in tandem with Iba-1 as a pan-macrophage marker as a measure of the polarisation state of microglia and monocytes at the lesion site (Fig. 7A–F). At 28 dpi, we found no significant difference in the overall number of CD86^+^ or Arg-1^+^ microglia or monocyte-derived macrophages, or in the relative proportion of CD86^+^ or Arg-1^+^ cells within the microglial or macrophage populations (Fig. 7G–I, S4). Intensity analysis of CD86^+^ cells revealed a decrease in CD86 expression in microglia following HGIL13 treatment compared to PBS-treated controls, however no difference was observed in CD86 expression in monocyte-derived macrophages between groups (Fig. 7H). Intensity analysis of Arg-1^+^ cells showed that Arg-1 expression was increased following HGIL13 treatment in both microglia and monocyte-derived macrophages compared to PBS- and HGBLANK- treated controls (Fig. 7J). Furthermore, when the ratio of CD86^+^ cells to Arg-1^+^ cells was compared across groups we found that the ratio of CD86^+^ to Arg-1^+^ microglia was decreased following HGIL13 treatment compared to PBS-treated controls, while no significant effect was observed in monocyte-derived macrophages (Fig. 7K). Overall, these findings suggest that HGIL13 modulates inflammation by reducing CD86 expression in microglia and increasing Arg-1 expression in both microglia and infiltrating monocytes. Although the ratio of CD86^+^ to Arg-1^+^ cells indicates that the overall inflammatory microenvironment remains skewed towards a proinflammatory state, HGIL13 effectively reduces this imbalance.

### 3.8 HGIL13 reduces inflammatory gene expression after SCI

**Fig. 8:**
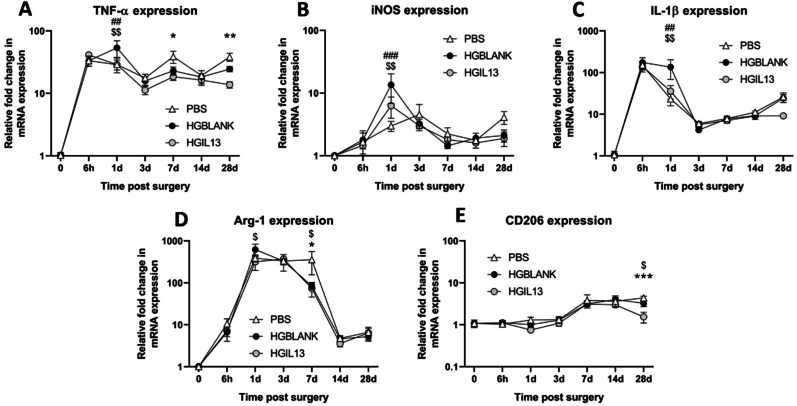
The temporal effect of HGIL13 on inflammatory gene expression after SCI. *Relative mRNA expression of (A) TNF-α, (B) iNOS, (C) IL-1β, (D) Arg-1 and (E) CD206 was determined at* 6 h*, 1, 3, 7, 14 and 28 dpi in wild-type C57BL6/J mice. Gene expression is represented as fold change compared to uninjured controls with PPIA as a housekeeping gene. Data represent mean ± SEM of n = 4-6 mice/group. Analysis by two-way ANOVA with Tukey's multiple comparisons test. ∗ represents a significant difference between PBS and HGIL13, # between PBS and HGBLANK, and $ between HGBLANK and HGIL13. ∗*^*,*^^*#, $*^*p < 0.05,*^*##, $$*^*p < 0.01, ∗∗∗*^*, ###*^*p < 0.001.*

The functional and histological results described thus far have focused on a 28-day timepoint to evaluate the therapeutic effect of HGIL13 on recovery after SCI. However, the ultimate goal of this proposed hydrogel approach is to provide sustained IL-13 delivery from the time of administration throughout the secondary injury phase. To assess the temporal response to IL-13 release, we measured inflammatory gene expression at the lesion site using RT-qPCR in wild-type C57BL6/J mice at six different timepoints post injury; namely 6 h, 1, 3, 7, 14 and 28 dpi (Fig. 8). We selected a panel of representative inflammatory markers - TNF-α, iNOS, IL-1β, Arg-1 and CD206 [11,60,61]. HGBLANK treatment increased TNF-α, iNOS and IL-1β expression at the lesion site at 1 dpi, however HGIL13 mitigated this increase (Fig. 8A–C). Furthermore, HGIL13 reduced TNF-α expression compared to PBS controls at 7 and 28 dpi (Fig. 8A). Arg-1 expression was significantly higher following HGBLANK treatment compared to HGIL13 at 1 dpi, however this effect was not sustained over time (Fig. 8D). CD206 expression was decreased in HGIL13-treated animals at 28 dpi (Fig. 8E). Notably, the expression of all analysed genes remained elevated at every timepoint post-injury compared to naïve uninjured controls indicating that secondary inflammation persists up to at least 28 dpi. Collectively, these results suggest that HGIL13 protects against HGBLANK-mediated inflammation at 1 dpi, with further modulation of TNF-α expression at later timepoints.

### 3.9 HGIL13 downregulates cell division in microglia at 7 dpi

To investigate the molecular mechanisms underlying improved recovery in HGIL13-treated mice, we analysed the transcriptomic profiles of microglia isolated from Hexb^tdTomato^ mice at 7 dpi using RNA-sequencing. This timepoint was chosen based on observations of improved BMS scores (Fig. 3A) and decreased TNF-α expression (Fig. 8A), both indicating the efficacy of HGIL13 by 7 dpi. Microglia (tdTomato^+^ cells) were successfully isolated via FACS with a yield of 25,000–78,000 microglia per sample (Fig. S5).

**Fig. 9:**
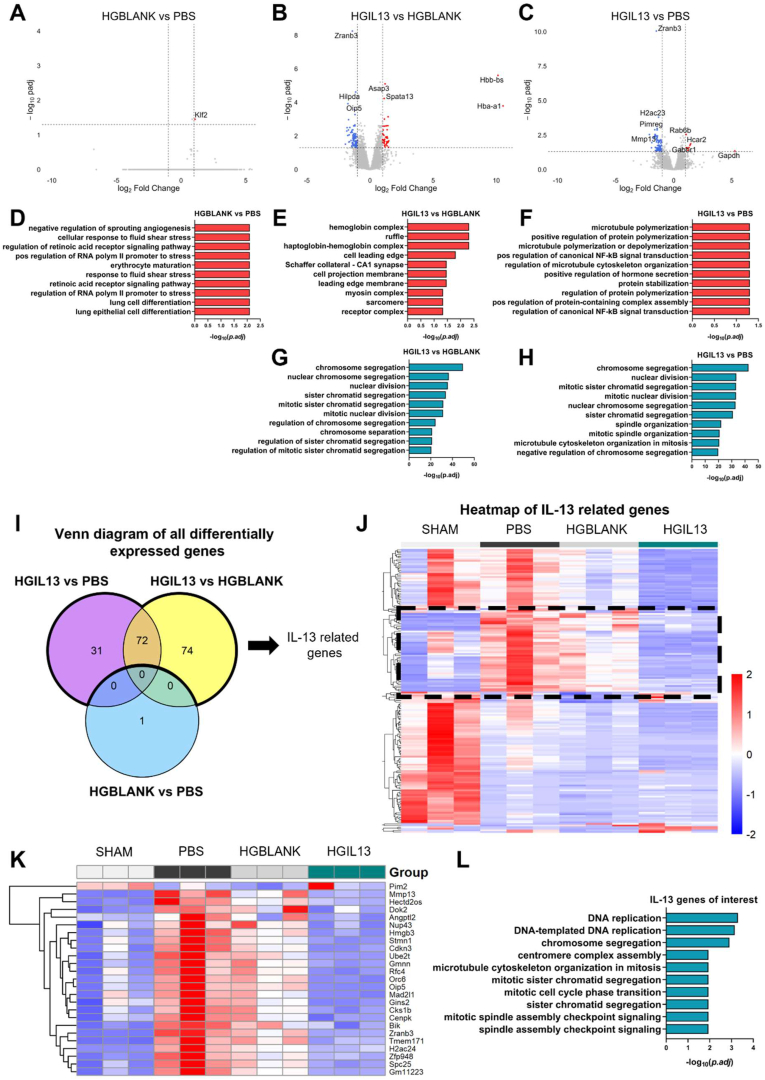
HGIL13 downregulates cell division in microglia at 7 dpi. (A–C) Volcano plots showing DEGs in microglia in (A) HGBLANK vs PBS, (B) HGIL13 vs HGBLANK and (C) HGIL13 vs PBS. Dotted lines representing DEG criteria of |log2FoldChange| ≥ 1 and padj ≤0.05. (D–H) Top 10 most significant (D–F) upregulated and (G–H) downregulated biological processes from gene ontology analysis of DEGs. (I) Venn diagram showing the number of unique and shared DEGs between pairs of groups. IL13-related genes can be identified by combined DEGs between HGIL13 vs PBS and HGIL13 vs HGBLANK, and removing DEGs shared by HGBLANK vs PBS. (J) Heatmap of IL-13 related genes, Scale = Z scores of read counts. Genes of interest that are upregulated by SCI and subsequently downregulated by HGIL13 are outlined by dotted black line and shown in more detail in (K). (L) Top 10 most significant biological processes from gene ontology analysis of IL-13 related genes from (K). N = 3 mice/group.

An overview of differential expression analysis of RNA-seq data obtained from each treatment group is provided in Fig. 9. Pairwise comparisons between each treatment group and non-injured Sham controls demonstrated that contusion SCI at 7 dpi leads to significant transcriptomic changes in microglia. Notably, pathways associated with mitochondrial respiration such as ‘oxidative phosphorylation’ and ‘mitochondrial respiratory chain assembly’ were upregulated, while pathways related to cytoskeletal reorganization, including ‘regulation of GTPase activity’ and ‘chromatin remodelling’, were downregulated (Fig. S6). Minimal differences were observed between microglia from HGBLANK and PBS groups, indicating a limited effect of hydrogel treatment alone on the microglial transcriptome post-injury (Fig. 9A–D). This aligns with the lack of functional improvement observed in the HGBLANK group.

In contrast, HGIL13-treated microglia exhibited significant transcriptional changes compared to other groups with an overall higher number of downregulated DEGs than upregulated DEGs (Fig. 9B and C). Although some distinct processes were upregulated in HGIL13 versus HGBLANK microglia compared to HGIL13 versus PBS microglia, there was a similar upregulation of processes involved in cell motility, such as ‘cell leading edge’, ‘cell projection membrane’, and ‘microtubule polymerization’ (Fig. 9E and F). The overall main effect of HGIL13 on microglia was the downregulation of cell division processes including ‘chromosome segregation’, ‘nuclear division’ and ‘sister chromatid segregation’ (Fig. 9G–H).

The Venn diagram in Fig. 9I illustrates the number of unique and shared DEGs between treatment groups. In total, 177 genes were differentially expressed by HGIL13 microglia (purple and green circles). Using a heatmap to visualise these IL-13-related genes (Fig. 9J), we observed a cluster of 25 genes that are upregulated in PBS and HGBLANK microglia compared to non-injured Sham animals and subsequently downregulated by HGIL13 treatment. These genes are listed in Fig. 9K. Gene ontology analysis of this list revealed that IL-13 downregulates processes involved in cell division such as ‘DNA replication’, ‘chromosome segregation’ and ‘centromere complex assembly’ in microglia at 7 dpi. Taken together, these data indicate that HGIL13 treatment modulates microglial function by downregulating cell division by 7 dpi and that this may contribute towards the beneficial effect of HGIL13 on recovery after contusion SCI.

## 4 Discussion

The secondary neuroinflammatory response after SCI remains a formidable challenge and controlled delivery of immunomodulatory agents presents a desirable therapeutic strategy for regeneration. Our study addresses this therapeutic need by designing a hybrid biomaterial system, integrating PLGA microparticles within a GelMA hydrogel matrix, tailored for the sustained release of IL-13 in a mouse contusion SCI model. We have shown that the HGIL13 delivery system significantly improves functional and histopathological recovery over 28 days, demonstrating the benefits of a biomaterial-based delivery system for sustained and localised IL-13 delivery in SCI.

The choice of a photocrosslinkable 3 % *(w/v)* GelMA hydrogel was based on our previously published work which demonstrated that this hydrogel formulation is biocompatible with low immunogenicity [37]. Building upon this work, we encapsulated IL-13 in PLGA microparticles and embedded these microparticles in GelMA hydrogel to achieve the HGIL13 delivery system. Accurate determination of encapsulation efficiency is important when looking towards translational relevance and dosage calculations. Various methods of direct protein extraction from PLGA microparticles have been described, including dimethyl sulfoxide extraction [62], dichloromethane extraction [63] and NaOH hydrolysis [40,45]. Important factors to consider when selecting an appropriate method are downstream quantification methods (i.e. ELISA, bicinchoninic acid assay), compatibility of extraction solvents with the selected assay, and the correct use of standard diluent to match the selected extraction method. We found that NaOH hydrolysis with subsequent neutralisation with HCl produced an aqueous sample that was compatible with ELISA (Section 2.3). Based on these results, we calculated the total dose of IL-13 as 12.2 ng per animal. Although this is lower than therapeutic doses reported in the literature (e.g. 100 ng intraspinal [24], 500 ng intraperitoneal [25]), we found that this dosage regime of HGIL13 improved functional recovery in a mouse contusion SCI model and highlights the advantage of continuous, localised delivery whereby a lower dose can achieve a functional effect directly at the lesion site. In fact, several studies have demonstrated that a higher dose of IL-13 may be neurotoxic. Van Broeckhoven et al. reported that while 5 ng/mL IL-13 reduced cell death in hippocampal slices *ex vivo,* 500 ng/mL IL-13 significantly increased cell death [28]. Similarly, Li et al. demonstrated that 50 ng/mL IL-13 could significantly reduce glutamate induced neuronal toxicity in vitro, but 450 ng/mL had no effect [64]. These findings suggest that a consistent supply is more important than a high dose of IL-13 when a neuroprotective effect is desired.

Release of IL-13 from the GelMA hydrogel alone exhibited a burst profile within the first 24 h, followed by minimal release over subsequent weeks due to depletion of readily diffusible IL-13 (Fig. 2A). In contrast, PLGA microparticles enabled sustained IL-13 release for up to 6 weeks *in vitro*. To achieve a more prolonged release, we combined both components in the HGIL13 formulation, leveraging the rapid release from the hydrogel and the extended release from PLGA microparticles. This dual-phase release profile featured an initial burst reflecting diffusion from the hydrogel matrix, followed by a sustained phase mediated by gradual microparticle degradation (Fig. 2A). Interestingly, an ‘expected release’ profile obtained from summing the amount of IL-13 released from each of the individual GelMA and PLGA components predicted a higher burst release over the initial 6 h than was observed experimentally (Fig. S1). This shows that the burst is limited in HGIL13, potentially enhancing drug retention for later timepoints. The cumulative release of IL-13 from HGIL13 at 6 weeks *in vitro* was 186 ± 18 pg IL-13/μL which accounts for less than 10 % of the total amount of IL-13 loaded in the sample, suggesting that the remaining IL-13 may yet to be released from degrading microparticles. In fact, some intact microparticles were observed after 28 days of incubation *in vitro* (Fig. 1H). IL-13 was detectable at the lesion site of HGIL13-treated animals 6 h post-surgery (Fig. S2A), with immunomodulatory effects on cytokine expression from 1 to 28 dpi (Fig. 8). Fluorescent microparticles remained in the lesion site at 28 dpi (Fig. S2B), consistent with the timeline of PLGA degradation *in vitro*. Together, these findings support the notion that IL-13 release from HGIL13 continues throughout the duration of the study.

The bioactivity of the released IL-13 was confirmed by showing the ability to reduce LPS-induced increases in TNF-α and iNOS, and by preventing the LPS-induced decrease in Arg-1 *in vitro* (Fig. 2). These effects were equal to that of pure recombinant IL-13, proving that IL-13 remains bioactive throughout the HGIL13 synthesis and release process. Despite the effect of HGIL13 on TNF-α and iNOS expression, there was no observed effect on IL-1β expression either *in vitro* or *in vivo*. This may be due to the more complex and inflammasome-dependent maturation and secretion of IL-1β [65]. While IL-13 can modulate NF-κB-driven genes via STAT6 signalling, IL-1β regulation is more complex and the STAT6-independent maturation and cleavage of IL-1β may be less sensitive to IL-13 intervention [20].

We then assessed the therapeutic efficacy of HGIL13 in the clinically relevant mouse contusion SCI model. The beneficial effects of IL-13 on functional recovery in preclinical SCI have been demonstrated in studies that achieved sustained IL-13 delivery either through cell-based delivery or repeat injections of recombinant protein [17,25,28]. However, cell-based delivery approaches carry the disadvantage of poor cell graft localisation and survival [31], while repeated intraspinal injections of IL-13 alone has translational limitations in terms of patient comfort and well-being. In contrast, a single administration of IL-13 alone has limited therapeutic effect likely due to the short cytokine half-life [24]. Repeated intraperitoneal administration of IL-13 in a mouse hemisection SCI model improved BMS scores at 28 dpi [25]. However, IL-13 is known to contribute to the pathology of allergic asthma and thus systemic administration should be avoided [66]. Biomaterial-based delivery approaches overcome these limitations by providing sustained and localised therapeutic release, as demonstrated in this work. Previous work by our group investigated cell-based delivery of IL-13 in a mouse hemisection model [17,28], however the contusion model more accurately mimics the complex injury mechanism seen in most human traumatic SCI cases [67]. Here, we have shown that localised biomaterial-based delivery of IL-13 improves functional and histopathological recovery compared to PBS and HGBLANK controls after SCI in Hexb^tdTomato^ mice (Fig. 3). The 28 dpi timepoint was selected based on the primary study outcome of measuring functional recovery using the Basso Mouse Scale. In general, a BMS recovery curve shows increasing functional recovery from 3 to 14 dpi, followed by a plateau over subsequent weeks as animals reach their functional capacity [48]. Therefore, early experimental timepoints may not capture the full dynamics of functional improvement following treatment. The lack of locomotor recovery in HGBLANK animals indicates that the hydrogel treatment alone is not sufficient for recovery and that IL-13 is essential for functional improvement, which correlates with the observed decrease in lesion size and demyelinated area following HGIL13 treatment. HGIL13 treatment reduced astrogliosis at the lesion epicentre and in the perilesional area, indicating an IL-13-dependent reduction in astrogliosis that is not observed in HGBLANK animals (Fig. 3, Fig. 4). The glial scar is a major barrier to regeneration after SCI, and modulation of the growth-inhibitory environment in this region by HGIL13 may facilitate recovery [7,8].

We then investigated the neuroprotective effect of HGIL13 by assessing neuronal apoptosis at 28 dpi (Fig. 5). Although the overall number of neurons was unchanged by HGIL13 treatment, there was a significant reduction in the number of apoptotic neurons. Li et al. recently showed that neurons express a functional IL-13Rα1 indicating that IL-13 can act directly on neurons [64]. Van Broekhoven et al. showed that IL-13 did not protect against NO-mediated cell death of murine Neuro2A or human SH-SY5Y neuronal cell lines despite the presence of IL-13Rα1 and IL-4Rα on these cells. However, in a more complex *ex vivo* brain slice model where both neurons and microglia were present, 5 ng/mL IL-13 was protective against NMDA-induced cell death [28]. Interestingly, IL-13 expression following LPS treatment of microglial cultures was only observed when neurons were present, but not in the presence of astrocytes or microglia alone [53]. When IL-13-neutralising antibodies were administered with LPS into the cerebral cortex of mice *in vivo*, iNOS and TNF-α expression were upregulated and this led to increased neuronal death [53]. Taken together, these data suggest that the mechanistic action of IL-13 relies on microglia/neuron interplay post-injury and that HGIL13 does not directly stimulate neurogenesis but rather it confers neuroprotection by indirectly limiting neuronal death, perhaps via microglia-mediated mechanisms.

It is well known that IL-13 induces an alternative activation state in microglia and monocyte-derived macrophages, classified by increased Arg-1 expression [17,26,28,68]. Previous studies have suggested that this polarising capacity is more pronounced in monocyte-derived macrophages than in microglia, since IL-13 increases the number of Arg-1^+^ macrophages but decreases the overall number of microglia at the lesion site in a hemisection SCI model [17]. Despite the strong polarisation potential of microglia *in vitro*, this suggests that there may be some factors present after injury that limit the polarisation capacity of microglia *in vivo*. Previous studies investigating the effect of IL-13 on neuroinflammation often failed to distinguish between microglia and monocyte-derived macrophages, instead opting to use markers such as Iba-1 [27,28] or CD11b [53,69] that identify both cell types to varying degrees. Some groups used a flow cytometric approach whereby microglia are labelled as CD11b ^+^ CD45^int^ and monocytes as CD11b ^+^ CD45^high^ [24]. However, microglia can upregulate CD45 expression in response to inflammatory stimuli and therefore become indistinguishable from peripheral macrophages [70]. Transgenic CX_3_CR1^eGFP/+^CCR2^RFP/+^ mice are commonly used to differentiate between green fluorescent protein (GFP)^+^ microglia and red fluorescent protein (RFP)^+^ monocytes [17,26,71]. However, peripheral macrophages also express CX_3_CR1 and can downregulate CCR2 expression over time, which limits the effectiveness of this model for monitoring microglia and monocyte-derived macrophages in central nervous system (CNS) trauma [17,72]. In comparison, the Hexb^tdTomato^ mouse line reliably and stably labels microglia, even under pathological conditions [43]. Using Iba-1 in conjunction with the Hexb^tdTomato^ mouse line facilitates reliable differentiation between microglia and peripheral monocytes, allowing for confident determination of the differential effects of IL-13 on these two cell types. An overview of the general Iba-1^+^ immune microenvironment showed that HGIL13 decreased the overall number of immune cells at the lesion site and that this was due to a reduction in the number of microglia rather than monocyte-derived macrophages (Fig. 4, Fig. 6F-H). Microglial depletion by colony-stimulating factor-1 receptor (CSF1R) inhibitors worsens recovery after SCI, while subsequent withdrawal of these inhibitors allows repopulation of a homeostatic microglial population that improves recovery [34,[73], [74], [75], [76]]. Given that microglial polarisation by IL-13 *in vivo* has proven challenging [17], these studies suggest that reduction of the activated microglial population may instead provide an opportunity for a more proregenerative microglial phenotype to repopulate the lesion site and facilitate recovery. Peripheral macrophages, in contrast, show strong polarising capacity *in vivo* and thus therapeutic efforts can focus on driving alternative activation rather than reducing macrophage numbers [17,24,26].

Analysis of CD86 and Arg-1 expression in microglia and monocyte-derived macrophages revealed no significant change in the overall number of CD86^+^ or Arg-1^+^ cells between groups, however the ratio of CD86^+^ cells to Arg-1^+^ cells was decreased following HGIL13 treatment, indicating that HGIL13 reduced the imbalance of the predominantly pro-inflammatory microenvironment (Fig. 7). The ratio of CD86^+^ to Arg-1^+^ immune cells generally increases over time post-SCI and a decreased ratio has been linked to reduced neuropathic pain [11,77]. HGIL13 decreased CD86 expression in microglia and increased Arg-1 expression in both microglia and infiltrating monocytes compared to PBS and HGBLANK controls. This indicates a shift towards an alternatively activated phenotype, which is consistent with the neuroprotective effects of IL-13 previously documented in CNS trauma models, where the benefits are largely mediated through microglia and macrophages rather than direct neuronal action [27,28,53]. Our previous work demonstrated that IL-13 treatment reduces detrimental contacts between activated microglia and damaged axons in a mouse hemisection SCI model [17,28]. Additionally, IL-13 enhances phagocytosis and efferocytosis in microglia and infiltrating monocytes which is crucial for effective tissue remodelling and wound healing [78,79]. Collectively, these findings suggest that HGIL13 treatment modulates immune cell polarisation via decreased CD86 expression in microglia and increased Arg-1 expression in both microglia and infiltrating monocyte-derived macrophages.

We then applied RT-qPCR to evaluate the inflammatory response at the lesion site over time. HGBLANK induced a transient increase in the proinflammatory markers TNF-α, IL-1β and iNOS at 1 dpi (Fig. 8). While our previous work showed that GelMA does not elicit an inflammatory response *in vitro* or *ex vivo* using BV2 microglia and organotypic mouse spinal cord slices [37], the complex SCI microenvironment—which includes local and infiltrating immune cells, neuron-glia interactions, myelin-associated inhibitors, CSPGs, and hypoxia—may exacerbate minor material-associated inflammatory effects not previously observed *in vitro* [4,5]. Moreover, the acidic degradation of PLGA can induce an inflammatory response in macrophages *in vitro*, with a more pronounced effect seen upon treatment with microparticles versus nanoparticles [80,81]. The presence of IL-13 within the biomaterial matrix prevented these acute and transient increases in TNF-α, IL-1β and iNOS expression. Although the translational relevance of the effect of HGIL13 versus HGBLANK treatment is limited, this does provide evidence for an acute burst release of IL-13 *in vivo*. Although no change in Arg-1 or CD206 gene expression was detected following HGIL13 treatment, Arg-1 expression was transiently increased in HGBLANK-treated animals at 1 dpi (Fig. 8D). Since Arg-1 competes with iNOS for the substrate arginine [82], this increase in Arg-1 expression may represent a competitive response to increased iNOS expression rather than a beneficial immune response to HGBLANK. Notably, the expression of all analysed inflammatory genes remained elevated at every timepoint post-injury compared to uninjured controls. This underscores the chronic nature of the inflammatory response in SCI and highlights the need for a sustained immunotherapeutic release system capable of long-term action against persistent inflammation. HGIL13 decreased TNF-α expression at 7 and 28 dpi compared to PBS controls, providing evidence for a multiphasic immunomodulatory effect. In a normal wound-healing response, the inflammatory phase lasts for 1–3 days [18]. However, the chronic inflammatory response in murine SCI features a second upregulation of TNF-α and IL-1β at 14–28 dpi which can drive a neurotoxic phenotype in astrocytes and exacerbate astrogliosis [60,83,84]. HGIL13 effectively counteracts this delayed proinflammatory response as evidenced by decreased TNF-α expression during this timeframe and a subsequent reduction in astrogliosis at 28 dpi (Fig. 3, Fig. 4).

To further investigate microglia-related mechanisms responsible for HGIL13-mediated recovery, we isolated tdTomato ^+^ microglia from Hexb^tdTomato^ mice at 7 dpi and conducted RNA-sequencing. Pairwise comparisons between each treatment group and non-injured Sham controls revealed that contusion SCI at 7 dpi induces substantial transcriptomic alterations in microglia. Specifically, we observed upregulation of pathways related to mitochondrial respiration, including ‘oxidative phosphorylation’ and ‘mitochondrial respiratory chain assembly’, alongside downregulation of pathways involved in cytoskeletal reorganization, such as ‘regulation of GTPase activity’ and ‘chromatin remodelling’ (Fig. S6). Mitochondrial oxidative phosphorylation is a major source of reactive oxygen species (ROS), and its dysregulation contributes significantly to secondary damage following SCI [[85], [86], [87]]. The downregulation of cytoskeletal remodelling processes may indicate altered microglial motility and responsiveness, as small GTPases are key regulators of microglial activation and morphological changes in response to injury [88,89]. These findings reflect the well-characterised timeline of microglial responses following SCI [6,18,90,91]. By 7 dpi, microglia are no longer in the early activation phase but have proliferated and adopted phenotypes that contribute to the secondary injury environment, including through oxidative stress and other neuroinflammatory mechanisms. Furthermore, GTPase activity is involved in neurite outgrowth and regeneration, and thus downregulation of these processes in microglia after SCI may reflect injury-induced neurodegeneration [92,93].

DEG analysis comparing HGBLANK to PBS microglia indicated that GelMA/PLGA alone had a limited impact on the microglial transcriptome. This aligns with the lack of functional and histopathological improvement observed at 28 dpi in the HGBLANK group. In contrast, HGIL13 had a significant effect on the microglial transcriptome at 7 dpi. Gene ontology analysis revealed enrichment of several biological processes related to cell migration and motility, including ‘ruffle’, ‘cell leading edge’, ‘cell projection membrane’, ‘microtubule polymerization’ and ‘regulation of microtubule cytoskeleton organization’ (Fig. 9E and F). These processes are central to microglial phagocytosis and efferocytosis, which enable microglia to migrate toward injury cues, clear apoptotic debris, and initiate resolution of inflammation to support tissue repair [73,78,94]. Our findings are supported by previous studies demonstrating a critical role for IL-13 in promoting efferocytosis. Proto et al. showed that IL-13 is required for macrophage-mediated clearance of apoptotic cells during inflammation resolution in mice [78], while Cai et al. identified the STAT6/Arg1 axis as a key regulator of microglial efferocytosis. In their stroke model, loss of STAT6 impaired dead neuron clearance and exacerbated functional deficits [94]. The increased expression of Arg1 in microglia and macrophages following HGIL13 treatment in our study is consistent with enhanced efferocytic activity and suggests a potential mechanism by which IL-13 promotes recovery after SCI. Moreover, we observed upregulation of gene ontology terms such as ‘hemoglobin complex’ and ‘haptoglobin-hemoglobin complex’ in HGIL13-treated microglia relative to the HGBLANK group (Fig. 9E). These complexes are known to stimulate CD163 expression, a scavenger receptor and known marker of alternative activation [[95], [96], [97]]. Notably, Peng et al. reported that CD163^+^ macrophage-derived small extracellular vesicles promoted functional recovery in a mouse contusion SCI model [98]. Together, these findings suggest that HGIL13 promotes an Arg-1^+^ phagocytic and reparative microglial phenotype, likely contributing to improved recovery through enhanced efferocytosis and inflammation resolution.

HGIL13 downregulated mitotic processes in microglia at 7 dpi, including ‘chromosome segregation’, ‘nuclear division’ and ‘mitotic sister chromatid segregation’ (Fig. 9G and H). Visualisation of IL-13-related genes on a heatmap revealed an interesting gene cluster that were upregulated following SCI and subsequently downregulated by HGIL13 treatment, thus presenting a potential mechanistic explanation for improved recovery by HGIL13 (Figure K). Gene ontology analysis of these genes confirmed that HGIL13 downregulated mitotic processes in microglia at 7 dpi (Fig. 9L). Previous studies have shown that IL-13 induces apoptosis in microglia at 3–4 days post stimulation and that subsequent reduction of the activated microglial population underlies the beneficial effect of IL-13 in the context of neuroprotection, rather than direct action of IL-13 on neurons [[53], [54], [55]]. It is therefore plausible that the observed reduction in cell division is due to early IL-13-induced apoptosis of microglia as this would explain the reduced number of microglia at 28 dpi (Fig. 6G). Evidence for the pro-apoptotic role of IL-13 in microglia is shown in Fig. 9C whereby glyceraldehyde-3-phosphate dehydrogenase (GAPDH) is upregulated in HGIL13 microglia. Although normally functioning as a glycolytic ‘housekeeping’ enzyme, it is now recognised that GAPDH functions in apoptosis and GAPDH expression is three times higher in apoptotic cells than in non-apoptotic cells [[99], [100], [101], [102]]. Specifically, S-nitrosylation of GAPDH by nitric oxide abolishes its catalytic activity and facilitates nuclear localisation where GAPDH is acetylated by p300/CREB binding protein (CBP) [99]. Downstream targets of p300/CBP become activated and cause cell death, e.g. by permeabilising the mitochondrial membrane during the intrinsic apoptotic pathway [103]. The mechanism of IL-13-induced apoptosis in microglia has been investigated previously by other groups [54,69]. Yang et al. showed that IL-13 upregulates expression of cyclooxygenase-2 (COX-2) in activated microglia and production of COX-2 prostaglandin (PG) products, including PGE_2_ and 15d-PGJ_2_ [54]. Binding of PGE_2_ to peroxisome proliferator-activated receptor (PPAR) γ induced death of activated microglia [54]. Furthermore, Berry et al. showed that IL-13 induced expression of CD36 in human monocytes via PPARγ activation by 15d-PGJ_2_, and CD36 plays a role in the recognition of apoptotic cells by phagocytes [104,105]. Given that arachidonic acid metabolism to PGE_2_ and 15d-PGJ_2_ by COX-2 acts as a potential source of ROS [18,106,107], this provides further evidence for the suggested mechanism whereby IL-13 enhances COX-2 activity, consequently upregulating ROS metabolism and inducing apoptosis in activated microglia. This downregulates mitotic processes, ultimately leading to an altered microglial population by 28 dpi. Given the knowledge that microglia are essential for repair after SCI [73], it is likely that the reduction in microglial numbers is not the key factor underlying the benefit of HGIL13 but rather an early reduction in the activated microglia population may provide the opportunity for a more efferocytotic phenotype, with reduced CD86 expression and increased Arg-1 expression, to repopulate the injury state and facilitate recovery [34,76].

While the induction of apoptosis in microglia is a likely therapeutic mechanism of IL-13, Fig. 9K highlights 24 genes whose expression is upregulated following SCI but reduced by HGIL13 treatment to levels comparable to those in Sham animals, potentially representing additional mechanisms of IL-13 in this model. Matrix metalloproteinases (Mmp), such as *Mmp-13*, are a family of proteolytic enzymes that are upregulated following SCI and contribute to breakdown of the blood spinal cord barrier, immune cell infiltration, and apoptosis [108,109]. Therapeutic inhibition of MMPs after SCI reduces edema, blood spinal cord barrier breakdown, neuropathic pain, and improves functional recovery [[110], [111], [112]]. We found that *Mmp-13* is downregulated in HGIL13 microglia, and Moriya et al. clarified the mechanism by which IL-13 regulates *Mmp-13* expression using human dermal fibroblasts [113]. They showed that IL-13-mediated *Mmp-13* downregulation occurs via PI3K/Akt3 and protein kinase C-δ signalling. Given the role of MMPs in ECM degradation, this suggests that HGIL13 may promote a proregenerative phenotype in microglia whereby ECM deposition and remodelling at the injury site is improved. Moreover, MMPs are downregulated in breast cancer cells following inhibition of high mobility group box-3 (*Hmbg3*), which was also identified as an IL-13 related gene in the current study. Several other of the identified genes have roles in regulation of remodelling (*Angptl2* [114]), inflammation (*Hectd2* [115], *Dok2* [116], *Stmn1* [117], *Orc6* [118], *Cks1b* [119]) and proliferation (*Nup43* [120]*, Cdkn3* [121], *Gmnn* [122], *Rfc4* [123]*, Mad2l1* [124]*, Gins2* [125]*, Bik* [126], *Zranb3* [127]*, Spc25* [128]). It is therefore likely that the beneficial mechanism of HGIL13 on microglia is multifaceted. Firstly, IL-13 induces apoptosis of the activated microglia early after injury, reducing the overall microglial population by 28 dpi. IL-13 then continues to act on this modified microglial population, which exhibit increased Arg-1 expression and decreased CD86 expression, by regulating remodelling and inflammatory pathways to ultimately improve functional and histopathological recovery.

Potential limitations of this study include the inability to precisely quantify IL-13 release *in vivo*. In pilot experiments, we measured IL-13 in spinal cord tissue from 6 h to 14 dpi using ELISA and immunofluorescently stained for IL-13 at 28 dpi. However, IL-13 levels were undetectable past 6 h post-surgery and no positive immunostaining was observed at 28 dpi (see Fig. S2 for 6 h data). Although there are some reports of measuring growth factor and antibody release from hydrogels *in vivo* by ELISA [129,130], the challenges of detecting recombinant cytokine release *in vivo* are extensive and not well discussed in the literature to date. In our experience, the compatibility of commercial IL-13 ELISA kits with recombinant IL-13 varies greatly and should be validated before undertaking this form of work. This highlights the technical limitation of using commercial antibody-based assays to detect recombinant cytokine release *in vivo*, where interference from other cells or proteins, cross-reactivity with other biomolecules, cytokine degradation or metabolization upon release, and temporal or spatial variability of cytokine release can all contribute to the difficulty of directly detecting IL-13 release [131,132]. We acknowledge the limited effect size observed in terms of the reduction in immune cell numbers at 28 dpi, whereby Iba-1+ immune cells are reduced from 632 ± 27 cells/mm^2^ in PBS mice to 520 ± 31 cell/mm^2^ in HGIL13 mice (Fig. 6). However, the improved locomotor capacity following HGIL13 treatment may not be fully due to the number of microglia but rather to the functional state of the more homeostatic microglia that repopulate the lesion site following early IL-13-mediated apoptosis of the activated population [34,76]. Additionally, the lack of significance observed in analysis of macrophage numbers and CD86/Arg-1^+^ cell counts may be due to the relatively large spread of the data points. However, we performed a quality check of the raw imaging data and the automated analysis pipeline to limit the influence of outliers on the analysis.

In this study, we present an exciting breakthrough in SCI repair with the first-ever demonstration of biomaterial-mediated IL-13 delivery in a preclinical setting. Our innovative HGIL13 system not only improved both functional recovery and tissue integrity over 28 days, but also reshaped the inflammatory microenvironment at the injury site. By reducing CD86 expression in microglia and boosting Arg-1 expression in both microglia and infiltrating monocyte-derived macrophages, we achieved a finely tuned immune response to foster tissue repair. Using transcriptomic profiling of Hexb^tdTomato^, we have uncovered a compelling mechanistic insight whereby HGIL13 downregulated mitotic processes in microglia at 7 dpi, ultimately reducing the microglial population by 28 dpi. These results emphasise the transformative potential of sustained cytokine delivery for SCI treatment and underscore the power of biomaterial-based strategies to drive recovery. The inclusion of neuroregenerative factors and integration with gold-standard rehabilitation opens an exciting new frontier in neuroregenerative medicine and introduces a promising option for next-generation SCI repair.

## CRediT authorship contribution statement

**Ciara M. Walsh:** Writing – original draft, Methodology, Investigation, Funding acquisition, Formal analysis, Data curation, Conceptualization. **Ruth Colbert:** Methodology, Investigation, Data curation. **James P. Reynolds:** Methodology, Investigation, Data curation. **Emily Dunne:** Methodology, Investigation, Data curation. **Emmanuelle D. Aiyegbusi:** Methodology, Investigation, Data curation. **Ross O'Carroll:** Methodology, Investigation, Data curation. **Jacek K. Wychowaniec:** Writing – review & editing, Methodology, Investigation. **Takahiro Masuda:** Methodology, Resources, Writing – review & editing. **Klaus-Peter Knobeloch:** Methodology, Resources, Writing – review & editing. **Marco Prinz:** Methodology, Resources, Writing – review & editing. **Dermot F. Brougham:** Writing – review & editing, Resources, Methodology, Funding acquisition. **Dearbhaile Dooley:** Writing – review & editing, Supervision, Resources, Project administration, Methodology, Investigation, Funding acquisition, Formal analysis, Conceptualization.

## Data availability statement

The data that support the findings of this study are available from the corresponding author upon reasonable request.

## Ethics approval and consent to participate

Procedures involving the use of animals were approved by the Animal Research Ethics Committee at University College Dublin (AREC-19-23) and the Health Products Regulatory Authority of Ireland (AE18982_P179) in accordance with the European Union Directive 2010/63/EU and S.I No. 543 of 2012.

## Declaration of competing interest

The authors declare no conflict of interest.
