# Supplementary material for: Localised delivery of interleukin-13 from a PLGA microparticle embedded GelMA hydrogel improves functional and histopathological recovery in a mouse contusion spinal cord injury model
Source: Bioact Mater. 2025 Aug 8;53:855–74. doi: 10.1016/j.bioactmat.2025.07.018 (PMC12355505; doi:10.1016/j.bioactmat.2025.07.018)
Supplement: Multimedia component 1 [file mmc1.docx]

**Supplementary material**


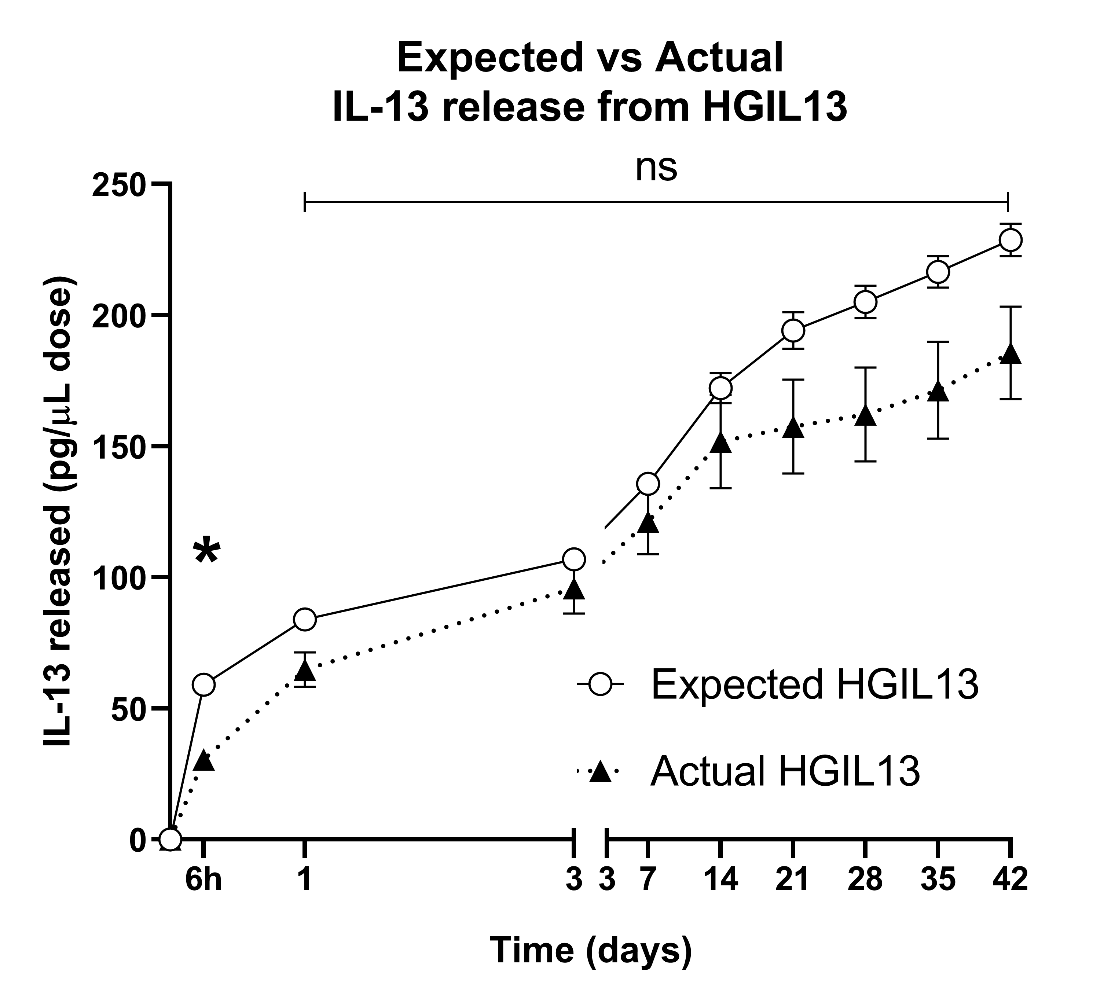


Figure S1: HGIL13 limits the expected burst release of IL-13 in the first 6 hr in vitro.

*The expected cumulative release of IL-13 from HGIL13 was calculated by summing the amount of IL-13 released at each timepoint from GelMA and PLGA, original data is shown in Figure 2A. Data represents mean ± SEM of n = 3 samples/group. Analysis by two-way repeated measures ANOVA with Bonferroni’s test for multiple comparisons, * p < 0.05, ns p > 0.05.*


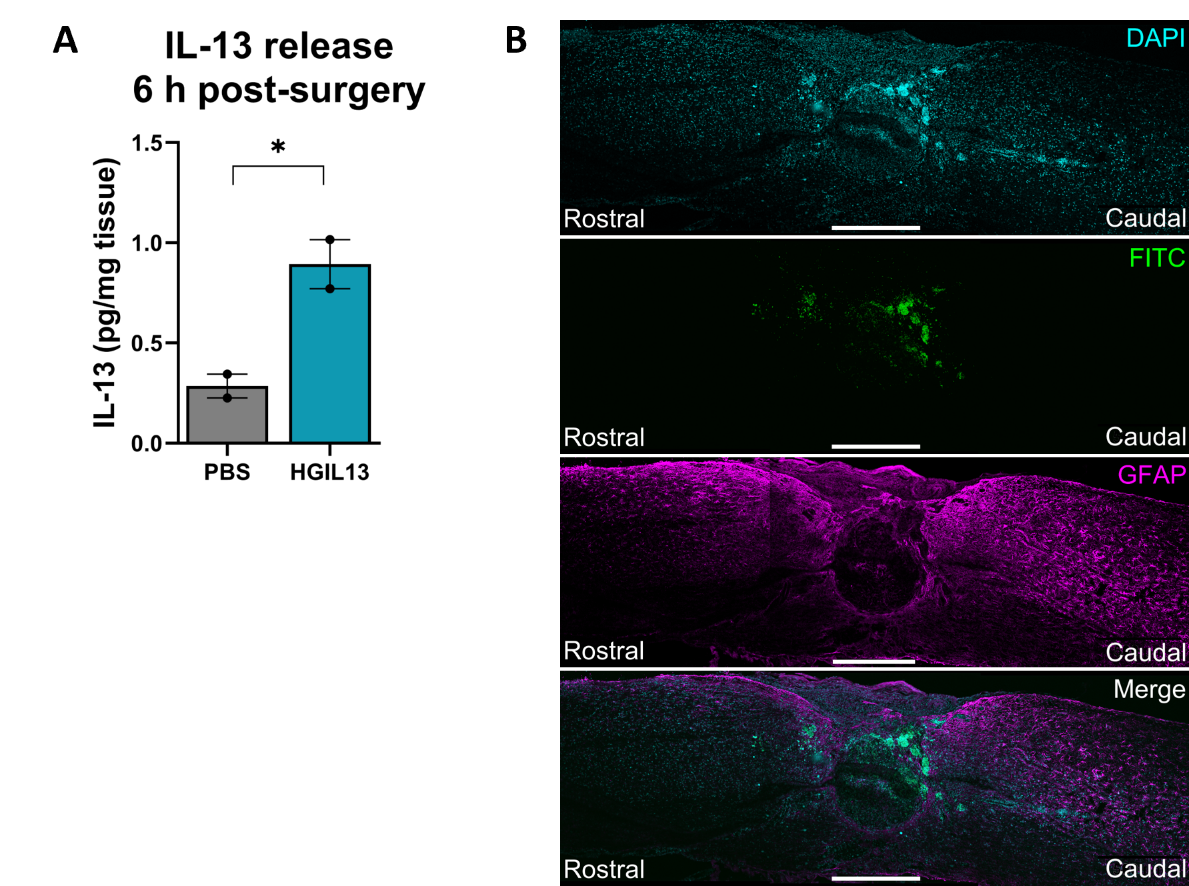


Figure S2: HGIL13 is distributed throughout the lesion site for localised delivery.

*(A) IL-13 is detectable at the lesion site of HGIL13 mice 6 h post-surgery, n = 2 mice/group. (B) Representative photomicrograph of a longitudinal spinal cord section 28 dpi depicting fluorescent microparticles (green, FITC) in the lesion which is identified by the glial scar (magenta, GFAP). Scale bar represents 500 µm. Analysis by unpaired Student’s t-test, * p < 0.05.*


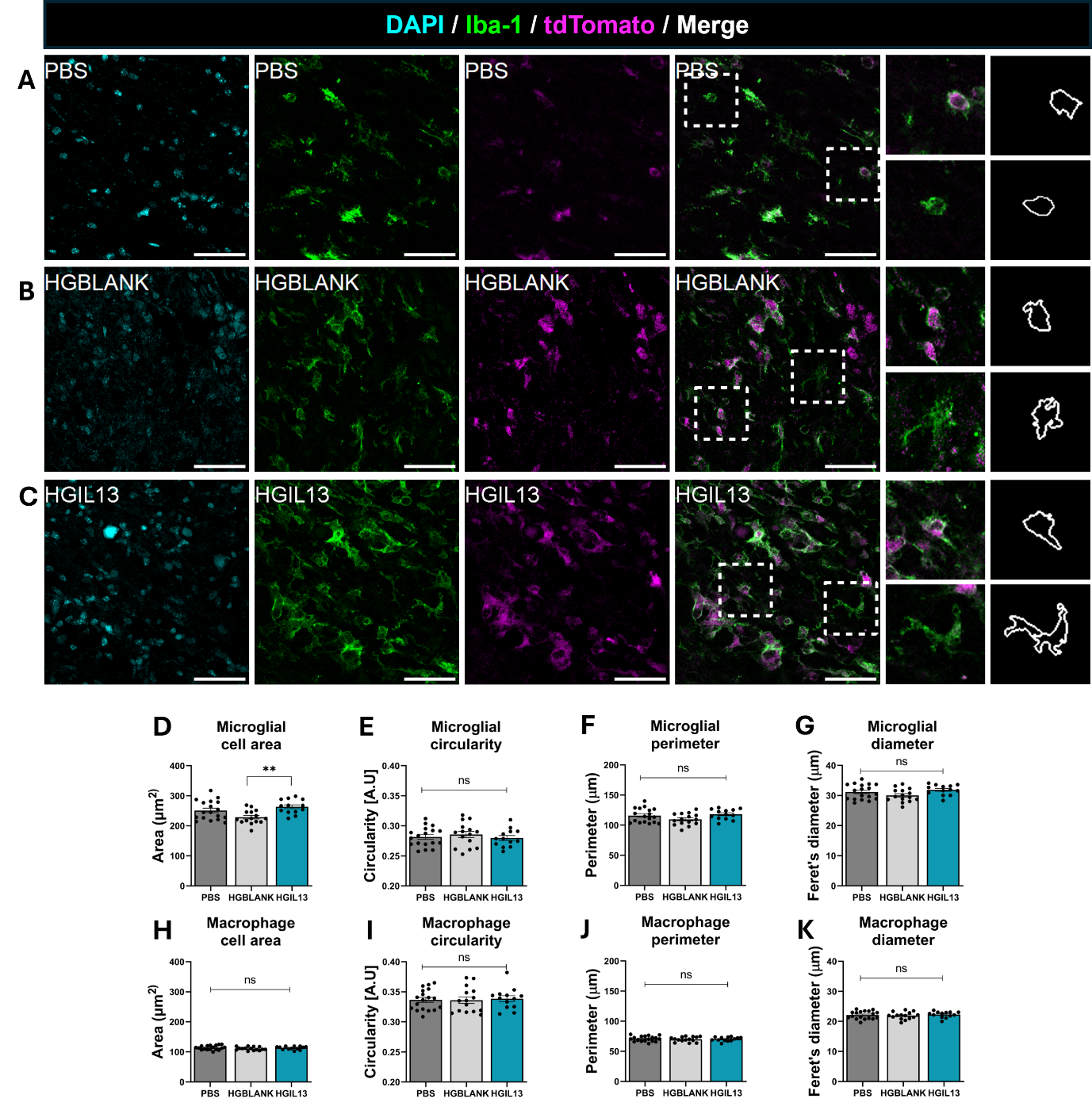


Figure S3: HGIL13 has no effect on the morphology of microglia or macrophages after SCI.

*(A-C) Representative photomicrographs of the lesion epicentre of Hexb^tdTomato^ mice after SCI showing Iba-1^+^tdTomato^+^ microglia and Iba-1^+^tdTomato^-^ macrophages. Examples of each cell type are highlighted by dotted white lines and their corresponding mask used for morphological analysis is shown on the right. Quantification of (D, H) area, (E, I) circularity, (F, J) perimeter or (G, K) diameter of microglia and macrophages was done using CellProfiler as described in Section 2.14. Scale bars represent 50 µm. Data represent mean ± SEM of 10,000 – 35,000 cells from n = 13-18 mice/group. Analysis by one-way ANOVA with Tukey’s multiple comparisons test, ** p < 0.01, ns p > 0.05.
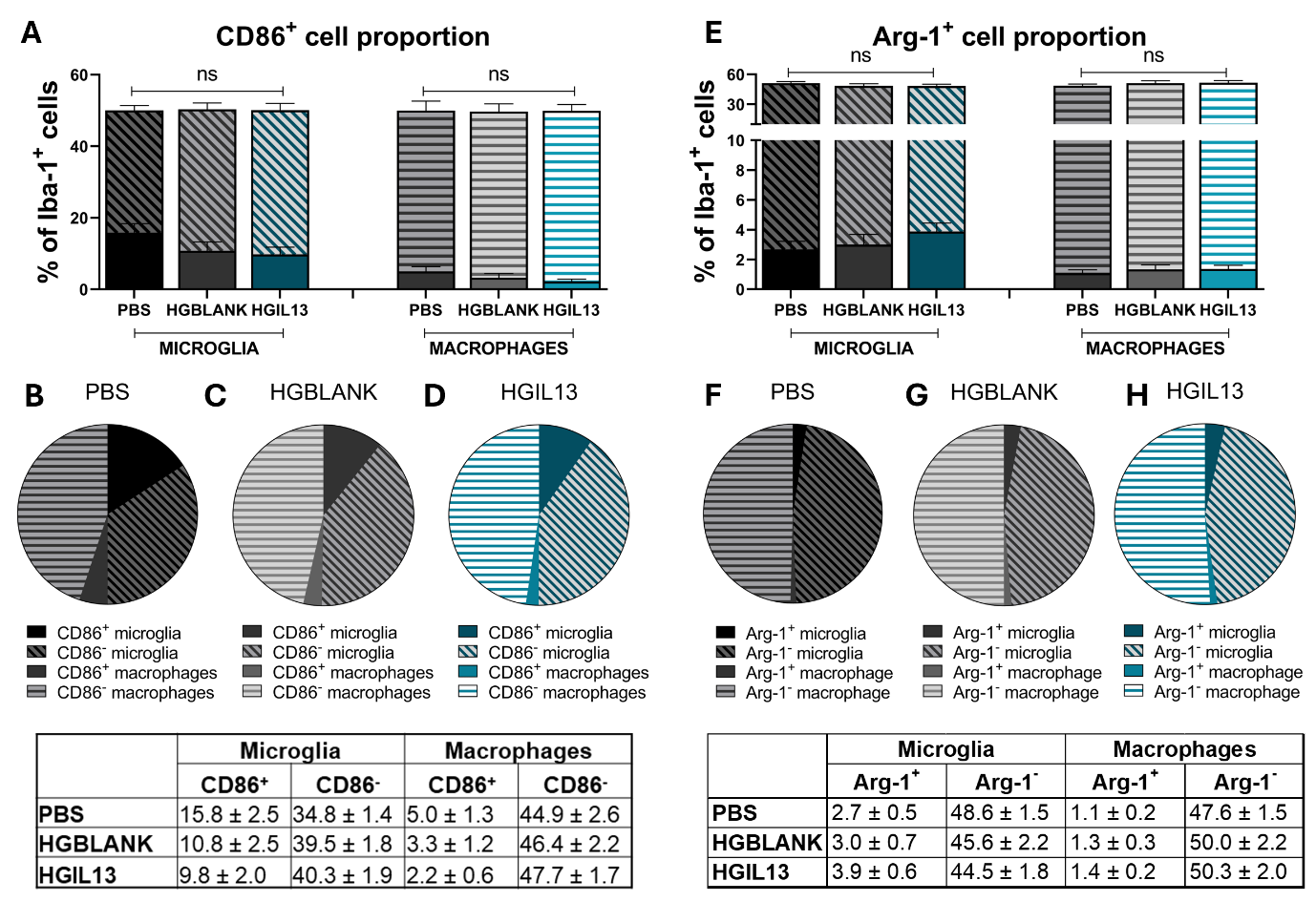
*

Figure S4: HGIL13 has no effect on the relative proportions of CD86^+^ or Arg-1^+^ immune cells after SCI.

*(A) Iba-1^+^tdT^+^CD86^+^ microglia and Iba-1^+^tdT^-^CD86^+^ macrophages and (E) Iba-1^+^tdT^+^Arg-1^+^ microglia and Iba-1^+^tdT^-^Arg-1^+^ macrophages were calculated as a proportion of the total Iba-1^+^ immune cell population in (B, F) PBS, (C, G) HGBLANK and (D, H) HGIL13 treated animals at 28 dpi. No significant difference was seen in the relative proportions of any cell populations between groups.* *Bar charts represent mean ± SEM of n = 13-18 mice/group, and pie charts represent relative proportions of Iba-1^+^ population* *with exact percentage details given in the table below. Analysis by Kruskal-Wallis with Dunn’s multiple comparisons test*, *ns p > 0.05.*

*
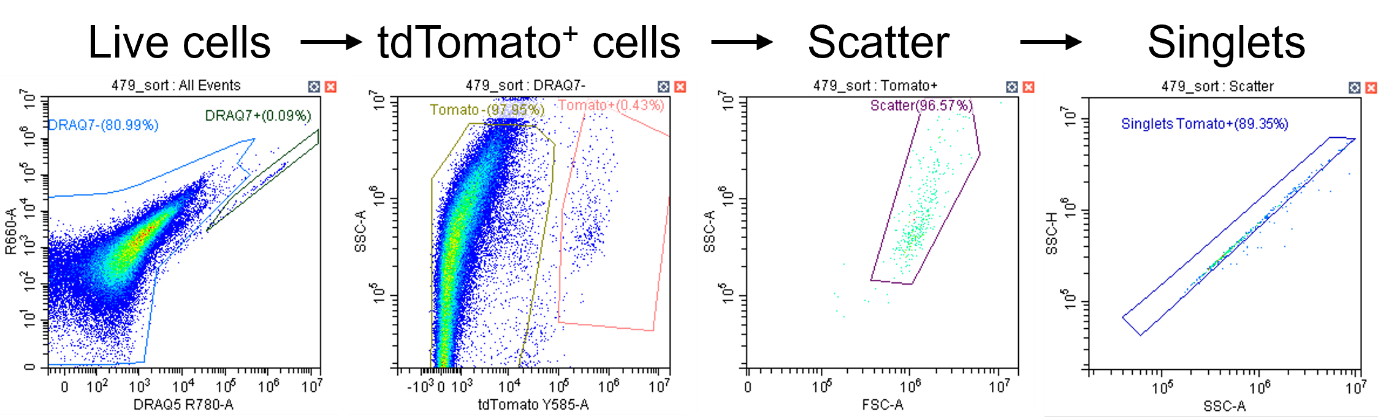
*

Figure S5: FACS gating strategy for sorting tdTomato^+^ microglia from Hexb^tdTomato^ mice.

*Live cells were identified as DRAQ7^-^. Gates were then established for tdTomato^+^ cells, cell scatter, and singlets.*

*
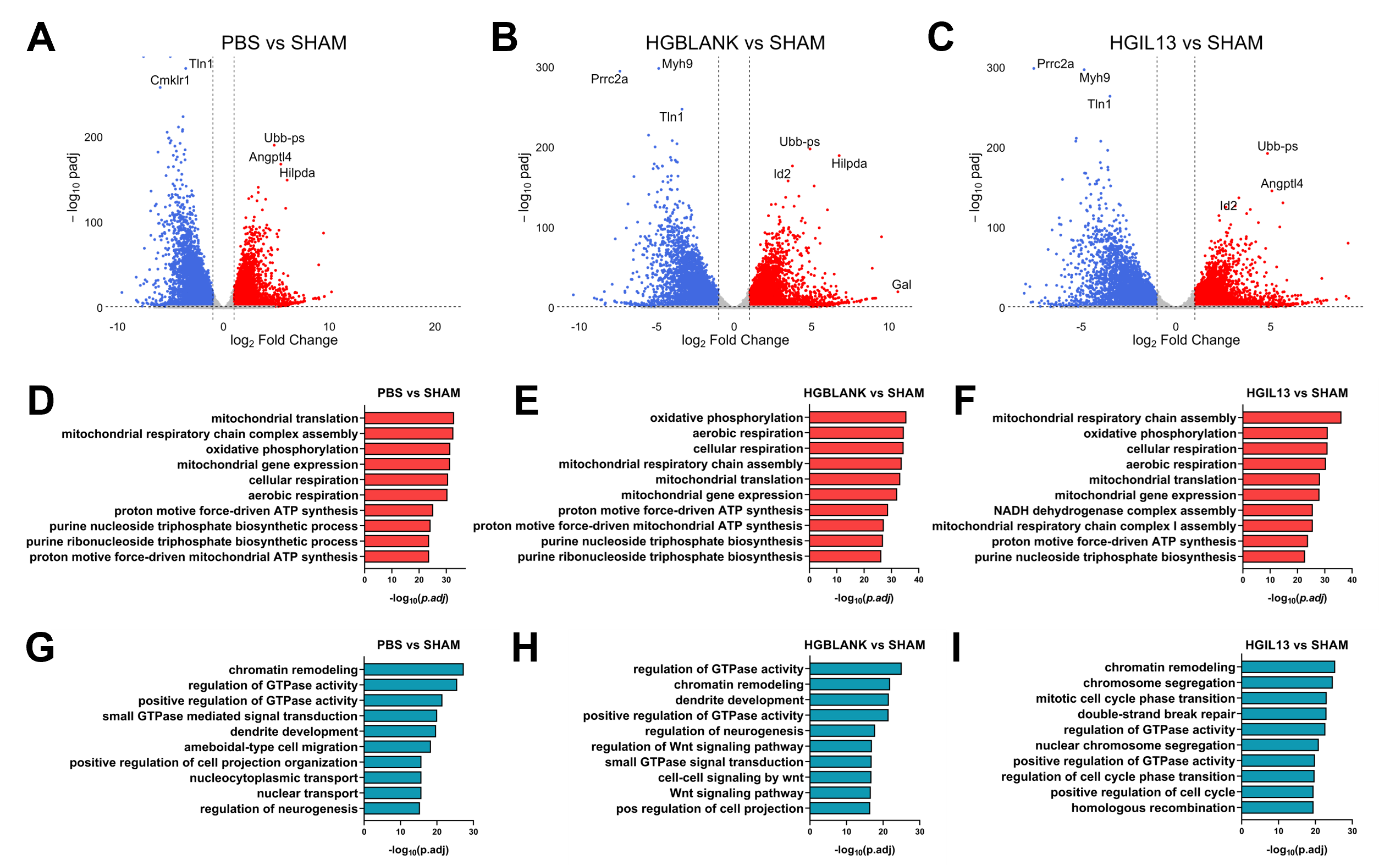
*

*Figure S6: DEG analysis of SCI groups versus Sham animals. (A-C) Volcano plots showing DEGs in microglia in (A) PBS vs Sham, (B) HGBLANK vs Sham and (C) HGIL13 vs Sham. Dotted lines representing DEG criteria of |log2FoldChange| ≥ 1 and padj ≤ 0.05. (D-I) Top 10 most significant (D-F) upregulated and (G-I) downregulated biological processes from gene ontology analysis of DEGs. N = 3 mice/group.*
